# L-Type Calcium Channels Play a Critical Role in Maintaining Lens Transparency by Regulating Phosphorylation of Aquaporin-0 and Myosin Light Chain and Expression of Connexins

**DOI:** 10.1371/journal.pone.0064676

**Published:** 2013-05-29

**Authors:** Rupalatha Maddala, Tharkika Nagendran, Gustaaf G. de Ridder, Kevin L. Schey, Ponugoti Vasantha Rao

**Affiliations:** 1 Department of Ophthalmology, Duke University School of Medicine, Durham, North Carolina, United States of America; 2 Department of Pathology, Duke University School of Medicine, Durham, North Carolina, United States of America; 3 Department of Biochemistry, Vanderbilt University School of Medicine, Nashville, Tennessee, United States of America; 4 Department of Pharmacology and Cancer Biology, Duke University School of Medicine, Durham, North Carolina, United States of America; University of Arkansas for Medical Sciences, United States of America

## Abstract

Homeostasis of intracellular calcium is crucial for lens cytoarchitecture and transparency, however, the identity of specific channel proteins regulating calcium influx within the lens is not completely understood. Here we examined the expression and distribution profiles of L-type calcium channels (LTCCs) and explored their role in morphological integrity and transparency of the mouse lens, using cDNA microarray, RT-PCR, immunoblot, pharmacological inhibitors and immunofluorescence analyses. The results revealed that Ca (V) 1.2 and 1.3 channels are expressed and distributed in both the epithelium and cortical fiber cells in mouse lens. Inhibition of LTCCs with felodipine or nifedipine induces progressive cortical cataract formation with time, in association with decreased lens weight in ex-vivo mouse lenses. Histological analyses of felodipine treated lenses revealed extensive disorganization and swelling of cortical fiber cells resembling the phenotype reported for altered aquaporin-0 activity without detectable cytotoxic effects. Analysis of both soluble and membrane rich fractions from felodipine treated lenses by SDS-PAGE in conjunction with mass spectrometry and immunoblot analyses revealed decreases in β-B1-crystallin, Hsp-90, spectrin and filensin. Significantly, loss of transparency in the felodipine treated lenses was preceded by an increase in aquaporin-0 serine-235 phosphorylation and levels of connexin-50, together with decreases in myosin light chain phosphorylation and the levels of 14-3-3ε, a phosphoprotein-binding regulatory protein. Felodipine treatment led to a significant increase in gene expression of connexin-50 and 46 in the mouse lens. Additionally, felodipine inhibition of LTCCs in primary cultures of mouse lens epithelial cells resulted in decreased intracellular calcium, and decreased actin stress fibers and myosin light chain phosphorylation, without detectable cytotoxic response. Taken together, these observations reveal a crucial role for LTCCs in regulation of expression, activity and stability of aquaporin-0, connexins, cytoskeletal proteins, and the mechanical properties of lens, all of which have a vital role in maintaining lens function and cytoarchitecture.

## Introduction

The human ocular lens needs to maintain its transparency and tensile properties over several decades to support normal vision. Any compromise in these characteristics can lead to impaired accommodation and cataract formation which is a global health burden. To achieve transparency, the ocular lens has evolved remarkable structural adaptations including an avascular phenotype, absence of organelles from mature fiber cells, radial packing of fiber cells, and an internal microcirculation system. [1], [2] Lens fiber cells also maintain high tensile strength to support deformability during visual accommodation. The differentiated and unusually long and thin fiber cells, which constitute the bulk of the lens, are derived from epithelial cells that exit the cell cycle at the lens equator. Subsequently, they embark on a differentiation process that induces extensive cell elongation, membrane changes, reorganization of the cortical cytoskeleton and cell adhesive complexes, and expression of various fiber cell abundant and specific proteins including the crystallins, cytoskeletal proteins and channel proteins. [3], [4], [5], [6] While the hexagonal geometry, ordered packing, deformability, membrane cytosketal network integrity and channel protein organization of fiber cells are considered critical for optical clarity and focusing ability of the lens, [1], [4] the molecular and biochemical mechanisms governing these unique structural specializations and interactions are not well understood.

Our recent work as well as work from other laboratories has documented the expression and distribution of several adaptor proteins in the lens with known roles in linking cytoskeletal proteins to membrane proteins, including the ERM (ezrin, moesin, radixin) proteins, ankyrin-B, NrCAM, periaxin, desmoyokin (AHNAK), beta-IV spectrin, dystroglycan, contactins and Caspr. [7], [8], [9], [10] Importantly, these scaffolding proteins have been shown to regulate membrane clustering and organization of ion channel proteins in neuronal and cardiac muscle cells. [11], [12], [13], [14] Further, the PDZ domain-containing protein AHNAK/desmoyokin has been demonstrated to interact directly and regulate LTCC activity, indicating a potential role for this protein in the organization of LTCCs in lens fibers as well. [15], [16], [17], [18] Given our long term research objective of determining the functional and regulatory significance of cytoskeletal scaffolding proteins involved in fiber cell membrane organization, and our limited understanding of the role of LTCCs in lens fibers, here we have undertaken an analysis of the expression and distribution profile of LTCCs in the lens to determine the role of these channel proteins in lens architecture and transparency.

Calcium homeostasis has been recognized to be critical for lens structural integrity and transparency, [19], [20] with elevated levels of lens calcium and the subsequent stimulation of calpain activity reported to cause cataract or lens opacification. [21], [22], [23], [24], [25], [26] Furthermore, intracellular calcium acting together with calmodulin has been reported to regulate the activities of the aquaporin-0 water channel and connexin gap junctions, as well as the stability of crystallin proteins which are required for lens transparency and architecture. [27], [28], [29], [30], [31], [32], [33], [34], [35], [36], [37] Therefore, a tightly regulated calcium influx is critical for homeostasis of lens physiology and function. Although there is indirect evidence for the presence of voltage-gated LTCCs in lens epithelial and fiber cells, limited knowledge is currently available regarding a direct role for these proteins in normal lens transparency, as well as their expression and distribution profiles within lens. [38], [39], [40], [41] Moreover, lens fibers have been reported to contain high levels of phosphorylated myosin light chain (MLC) relative to lens epithelium, with the phosphorylation of MLC being regulated by calcium/calmodulin-dependant MLC kinase. [42] Significantly, inhibition of MLC kinase activity has been demonstrated to induce lens opacification, indicating that biochemical pathways/mechanisms regulating MLC kinase activity, such as regulation of intracellular calcium flux and activity of calcium/calmodulin dependent MLC kinase, play an important role in lens function. [42] While the LTCCs are known to regulate intracellular calcium flux, their potential role in regulation of MLC phosphorylation, and the activities and expression of aquaporin-0 and connexins in the lens is not well understood.

Based on the significant role that intracellular calcium appears to play in the lens, in this study we determined how impairment of LTCC activity might influence lens transparency and fiber cell structural integrity. This study reveals that LTCC inhibition in ex-vivo mouse lenses impairs water permeability, cytoskeletal organization and transparency in association with dysregulated aquaporin-0 serine phosphorylation, myosin light chain phosphorylation and expression of connexins, and uncovers an essential role for LTCC activity in maintenance of lens transparency and function.

## Results

### Expression and Distribution of Ca (V) 1.2 and 1.3 Channels in Mouse Lens

**Figure 1:**
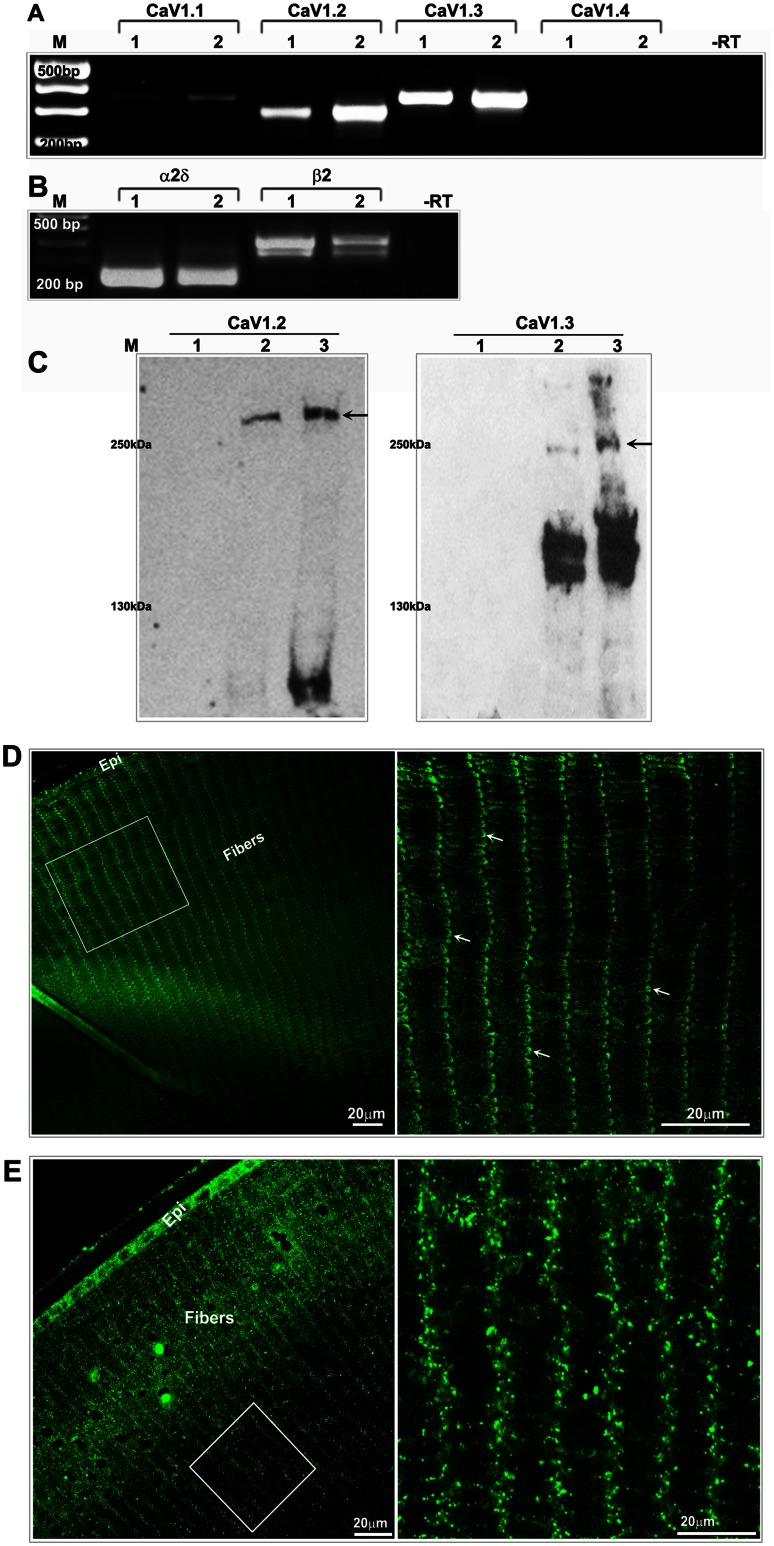
Expression and distribution of Ca (V) 1.2 and 1.3 channels in mouse lens. A and B. RT-PCR-based confirmation of expression of α1C (Ca (V) 1.2), α1D (Ca (V) 1.3), α2δ and β2 (auxiliary subunit genes) in P2 (lane 1) and P21 (lane 2) in mouse lenses. As shown in Panel A, the expression of Ca (V) 1.1 and 1.4 expression was found to be minimal relative to the expression profile of Ca (V) 1.2 and 1.3 in the same mouse lenses, under identical RT-PCR conditions. A –RT control was used in the RT-PCR experiments. B. Immunoblotting-based confirmation of the presence of Ca (V) 1.2 and 1.3 channel proteins in P21 mouse lens membrane enriched [Lanes 2 (75 µg protein) and 3 (125 µg protein)] fractions. The soluble lens fraction (Lane 1, 100 µg protein) did not exhibit immunopositive reactivity against the same antibodies. Arrows indicate the native Ca (V) 1.2 and 1.3 proteins. C. Distribution of Ca (V) 1.2 in P21 mouse lens equatorial sections based on immunofluorescence analysis. Right panel shows a magnified area (boxed in the left panel) and reveals the discretely clustered organization of Ca (V) 1.2 (arrows) at the short arms of the lens hexagonal fiber cell. D. Distribution analysis of Ca (V) 1.3 in P21 mouse lens equatorial sections reveals its intense localization to the short arm (right panel) of the hexagonal lens fiber cell. Bars: 20 µm. Epi: Epithelium.

To determine the expression of LTCCs in lens tissue, we utilized several approaches including cDNA microarray, RT-PCR and immunoblot analyses. cDNA microarray analysis of RNA derived from a 7-day old C57BL/6J mouse lenses revealed expression of different LTCC genes including Ca(v) 1.1, 1.2, 1.3 and 1.4 at varying levels, together with their auxiliary subunit genes α2δ and β (data not shown). Based on the cDNA microarray data, we examined for the expression profile of the pore forming subunit genes of all four LTCC subtypes including Ca (V) 1.1, 1.2, 1.3 and 1.4 along with the expression of their auxiliary subunits (α2δ and β) by RT-PCR analysis using RNA derived from P2 and P21 mouse lenses. As shown in Fig. 1A., the expression profiles of Ca (V) 1.2 and 1.3 were readily detectable compared to the expression of Ca (V) 1.1 and 1.4 which is consistent with the known wide range of tissue expression for Ca (V) 1.2 and 1.3 channels relative to Ca (V) 1.1 and 1.4, which exhibit restricted tissue distribution. [43], [44], [45], [46] Both P2 and P21 mouse lenses exhibited expression of the LTCC auxiliary subunit genes including α2δ and β2 as shown in Fig. 1B. Expression of Ca (V) 1.2 and 1.3 proteins was then assessed by immunoblotting analysis of membrane rich and soluble fractions derived from three week-old mouse lenses, using the appropriate monoclonal antibodies. As shown in Fig. 1C, Ca (V) 1.2 and 1.3 antibodies yielded specific immunopositive signals only in the membrane enriched lens fraction, identifying proteins of the expected molecular mass (∼ 250 kDa), together with proteins in the lower range of ∼ 160 kDa which represent truncated forms of LTCCs (as per antibody provider) confirming the presence of Ca (V) 1.2 and 1.3 channel proteins in the mouse lens. Intriguingly, the immunoreactivity with lower or fragmented proteins especially with Ca (V) 1.3 specific antibody was rather intense and the reasons for this are not clear. We also examined the distribution pattern of Ca (V) 1.2 and 1.3 channel proteins in the P21 mouse lens equatorial sections by immunofluorescence-based confocal imaging, which revealed that Ca(V) 1.2 and 1.3 channel proteins are distributed to the lens epithelium and fiber cells of the outer and inner cortical regions (Fig. 1D and E). In mouse lens equatorial sections, both Ca (V) 1.2 and 1.3 are localized predominantly to the short arms of hexagonally shaped fiber cells (Fig. 1D and E). Although both channel proteins are localized intensely to the short arms relative to the long arms of fiber cells, there appear to be some distinct differences in the subcellular distribution pattern at the short arms of the fiber cells. While Ca (V) 1.3 distribution along the short arm is localized uniformly to the plasma membrane, Ca (V) 1.2 channel protein exhibits a rather patchy and clustered distribution patter at the short arm of fiber cells (Fig. 1D and E, right panels). Although the use of both antibodies (Ca (V) 1.2 and 1.3) appears to be associated with some immunostaining in the cytosol of the lens epithelium, the soluble fraction of lens homogenates did not exhibit any immunopositive reactivity to these antibodies by blot analysis indicating some non-specific staining in the cytosolic regions of lens (Fig. 1D and E). These distribution patterns of LTCCs were confirmed in two independent lenses which were fixed at different times to rule out fixation variability. Since the lens nucleus is very hard, this region did not exhibit much immunopositive staining, as is the case for many other proteins examined.

### Inhibition of L-type Calcium Channel Activity Impairs Transparency in Ex-vivo Mouse Lenses

**Figure 2:**
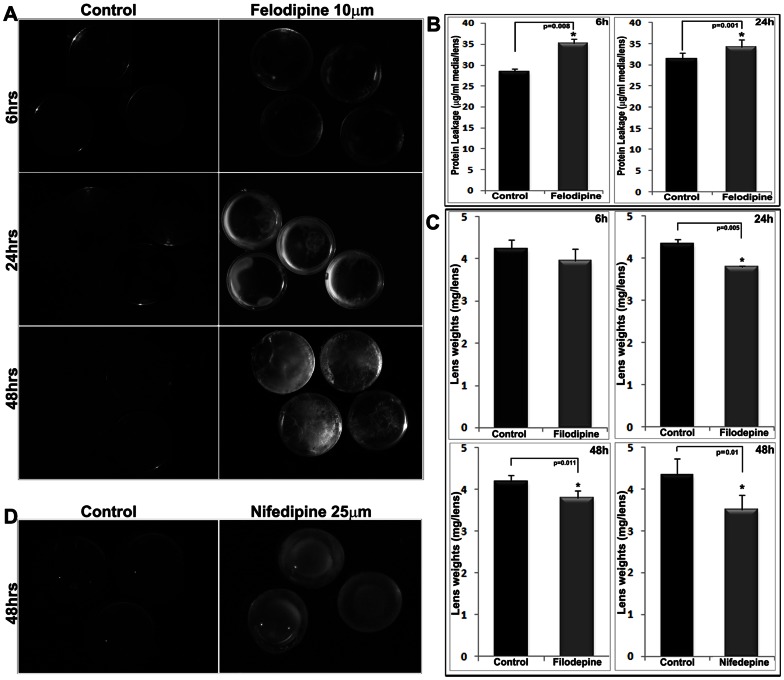
Felodipine and Nifedipine-induced lens opacification in ex-vivo cultured mouse lenses. A. Treatment of organ cultured (P21 to P26) mouse lenses with felodipine (10 µM) induced progressive lens opacity (subcapsular to cortical) with increasing time. Control lenses treated with DMSO (0.025%) remained transparent throughout the experiment. B. The felodipine treated lenses showed significant (* P<0.01) increase in protein secretion/leakage (µg/ml media/lens) into the media as compared to control lenses, based on the mean value from an n = 4 to 6 samples. D. Mouse lenses (P21 to P26) treated with nifedipine (25 µM) exhibited cortical and nuclear opacity after 48 hr of drug treatment, while control lenses treated with DMSO alone (0.025%) remained transparent. C. Both felodipine and nifedipine treated mouse lenses showed a significant (* P<0.01; n = 4–6) decrease in lens wet weight (mg/lens) compared to the corresponding controls.

To determine a potential role for LTCCs in maintenance of lens transparency, we examined the effects of dihydropyridine inhibitors felodipine and nifedipine, which are specific antagonists of LTCCs, on organ cultured mouse lenses. [44], [46], [47] Lenses derived from P21 to P26 day-old mice (male and female animals of the C57BL/6J strain) treated with 10 µM felodipine (dissolved in 0.025% DMSO) exhibited time dependent changes in transparency (Fig. 2A). While lenses treated with felodipine (10 µM) for 6 hrs remained mostly transparent with a few specimens exhibiting only a spotty subcapsular/cortical haziness or opacity, all lenses developed an intense peripheral ring-like subcapsular opacity after 24 hrs of exposure to drug. By 48 hrs of treatment, the opacity extended throughout the cortical region of lens (Fig. 2A). All cultured lenses exhibited protein leakage into the medium over the course of drug treatment, with the response being moderately (∼10 to 20%) but significantly higher in the drug treated lenses as opposed to control lenses (Fig. 2B). Protein leakage from drug treated lenses did not increase progressively with increasing length of drug exposure (Fig. 2B). Lenses treated with drug for either 6 or 24 hrs exhibited a comparable degree of (∼35 µg/ml of culture medium/lens) protein leakage. Intriguingly, however, the lens weight (wet) of drug treated specimens showed a significant decrease as compared to the control lenses after 24 and 48 hrs of incubation (Fig. 2C). Lenses treated with nifedipine (25 µM) which is known to be a less potent inhibitor of LTCC activity relative to felodipine, [47] exhibited no obvious opacity after 24 hrs of drug treatment (data not shown) but turned opaque with the opacity extending into the nucleus as well as the cortical region following a 48 hrs exposure to drug (Fig. 2D). Similar to the findings in felodipine treated lenses, treatment with nifedipine caused significant reductions in wet weight compared to control lenses after 48 hrs of exposure to drug (Fig. 2C). Control lenses were treated with appropriate volumes of vehicle (0.025% DMSO) used to dissolve drugs.

### L-type Calcium Channel Inhibition Induces Disorganization and Swelling of Lens Cortical Fiber Cells in Association with Alterations in Protein Profile

**Figure 3:**
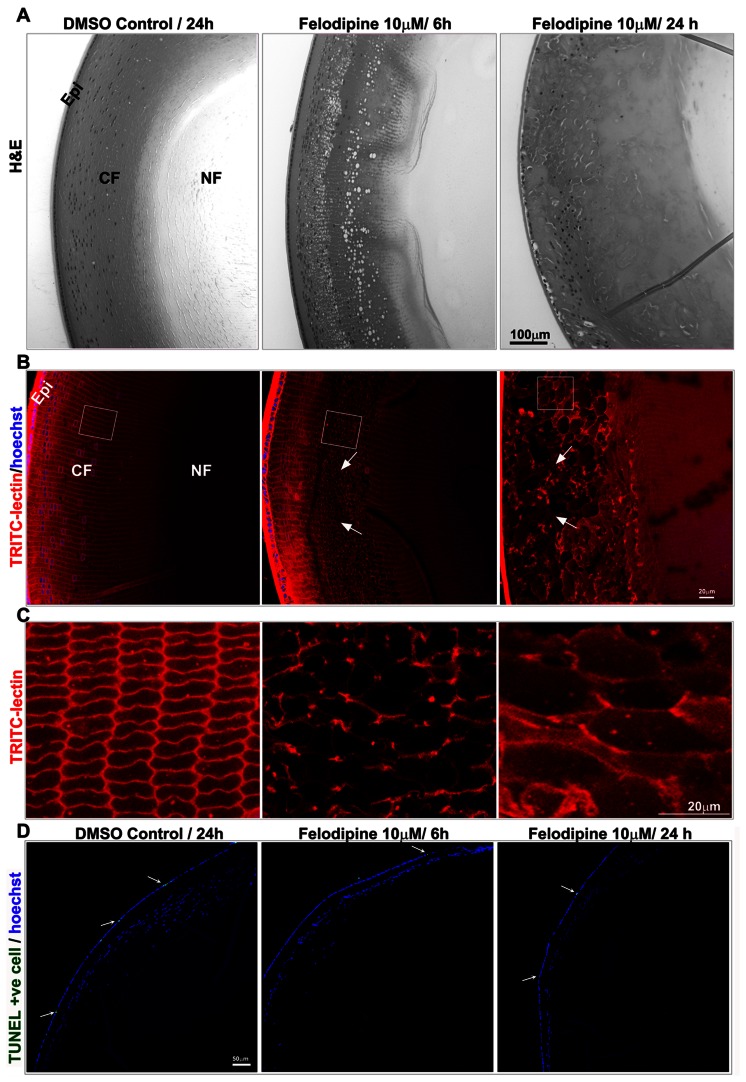
Inhibition of LTCCs by felodipine induces extensive disorganization and swelling of cortical fiber cells in ex-vivo organ cultured mouse lenses without any noticeable cell death. Treatment of organ cultured mouse lenses (P21 to P26) with felodipine (10 µM) induced progressive disorganization of cortical fiber cells (arrows) starting from 6 hrs of treatment. After 24 hrs of drug treatment, lens cortical fibers exhibited extensive swelling (see inserts and arrows) with large intracellular spaces based on light microscope examination (H&E staining) (A) and fluorescence microscopy analysis of equatorial plane tissue specimens using wheat germ agglutinin staining (B & C). The images in Panel C indicate the magnified area depicted in Panel B with squares. DMSO treated control lenses remained histologically intact at both 6 (not shown) and 24 hrs of incubation. D. TUNEL and Hoechst staining of felodipine treated (both 6 and 24 hr) and control lenses reveals comparable staining patterns for both the TUNEL positive (green cells indicated with arrows) and cell nuclei (in blue). Bars indicate magnification. Epi: Epithelium, CF: Cortical fibers, NF: Nuclear fibers.

Histological analysis of mouse lenses treated with felodipine (10 µM) for 6 hrs revealed an intact epithelium and outer cortical fibers, but extensive disruption of organization of inner cortical fiber cells (indicated with arrows; Fig. 3B) especially peripheral fibers of the inner cortical region, which displayed extensive swelling compared to control lenses (Fig. 3A, B and C). Extending the length of drug treatment to 24 hrs resulted in extensive disorganization of outer cortical fiber cells along with inner cortical fiber cells of the peripheral region with intact epithelium, and dramatically enhanced fiber cell swelling (Fig. 3 B, arrows) leading to larger intracellular spaces and a disruption in fiber cell organization (Fig. 3C shows a magnified area in panel B indicated with a square box). Interestingly, treatment of lenses with felodipine for either 6 or 24 hrs did not affect the deeper inner cortical fibers and the nuclear region fiber cells, which remained intact and exhibited compact organization similar to control lenses consistent with the lack of opacity observed in the nuclear regions as assessed by H&E staining (Fig. 3A) and WGA-based fluorescence staining (Fig. 3B and C). Drug-induced adverse effects or cytotoxicity was evaluated in lenses treated with felodipine for either 6hrs or 24 hrs by detecting apoptosis (green staining, arrows) and nuclei integrity (blue staining) and distribution using TUNEL and Hoechst staining, respectively (Fig. 3D). Both types of analyses confirmed the absence of drug-induced cytotoxic effects in felodipine treated lenses relative to control (DMSO treated) lenses (Fig. 3D).

**Figure 4:**
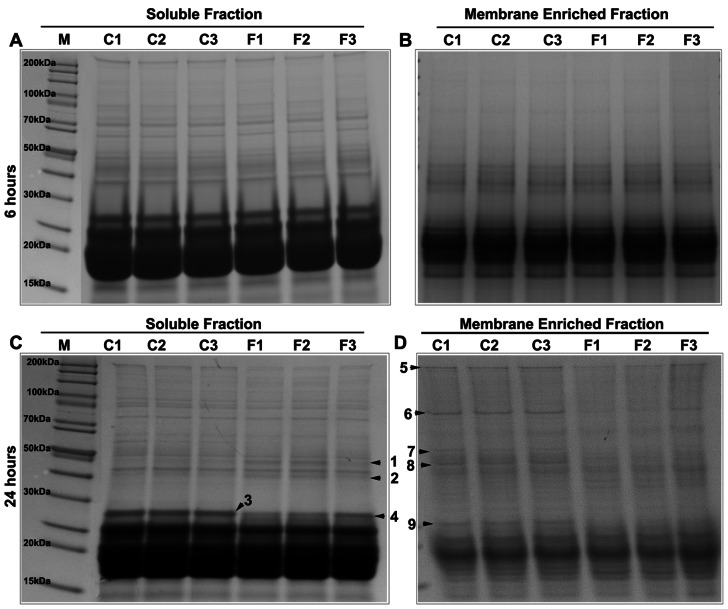
Felodipine-induced changes in protein profile of ex-vivo organ cultured mouse lenses. Felodipine (10 µM for 6 hrs or 24 hrs) and the corresponding DMSO treated control mouse lenses (p21 to P26) were homogenized and separated into soluble (100,000×g supernatant) and insoluble (membrane-enriched) fractions. Equal amounts of protein from soluble (A, C) and insoluble (B, D) fractions were then separated by gradient SDS-PAGE (4–20%) and stained with GelCode blue. Protein bands that exhibited obvious differences in staining intensity between the felodipine treated and control lenses (annotated using numbers and arrowheads in the gel images) were extracted, trypsinized and identified using a Synapt G2 mass spectrometer. See Table 1 for a listing of the proteins identified by mass spectrometry. C1 to C3 and F1 to F3 are individual specimens from control and felodipine treated lenses, respectively.

The above described histological alterations and protein leakage changes noted in felodipine treated mouse lenses (Figs. 2 & 3) led us to evaluate possible alterations in the protein profiles of lens soluble and insoluble fractions. These analyses were performed using SDS-PAGE in conjunction with GelCode blue staining of resolved proteins. As shown in Fig. 4, there were no obvious difference in the protein profile of soluble (A) and membrane-enriched insoluble fractions (B) in the 6 hrs felodipine treated lenses as compared to control lenses. However, marked differences were noted for selected proteins in both soluble (Fig. 4C) and insoluble fractions (Fig. 4D) of lenses treated with felodipine for 24 hrs. To identify proteins which exhibited obvious decrease in their band intensity relative to control samples based on SDS-PAGE analysis and gelcode blue staining of felodipine treated lenses, protein bands of interest (indicated with arrow heads) were extracted from SDS-PAGE gels, trypsin digested and subjected to mass spectrometry-based (Synapt G2) identification. The mass spectrometry analysis identified β-B1 crystallin, Hsp-90, filensin, β-catenin, phakinin, spectrin, 14-3-3ε and vimentin as proteins which exhibited decrease in levels in drug treated lenses (Table 1).

**Table 1:** The Felodipine treated mouse lenses exhibit decreased levels of certain selected proteins identified by Mass spectrometry.

Sample 1	Bfsp2 Isoform 1 of PhakininVimentin
Sample 2	Bfsp2 Isoform 1 of PhakininCytoplasmic 2 ActinVimentin
Sample 3	β- Crystallin B114-3-3 ε
Sample 4	β-Crystallin B114-3-3 ε
Sample 5	β-Spectrin
Sample 6	FilensinHSP 90ββ-Catenin
Sample 7	VimentinGap Junction α8
Sample 8	PhakininFilensin
Sample 9	β Crystallin B1β Crystallin B3

### Deregulated Aquaporin-0 Phosphorylation, Expression of Connexins and Decreased Levels of 14-3-3ε Protein Precedes Lens Opacity Induced by LTCC Inhibition

**Figure 5:**
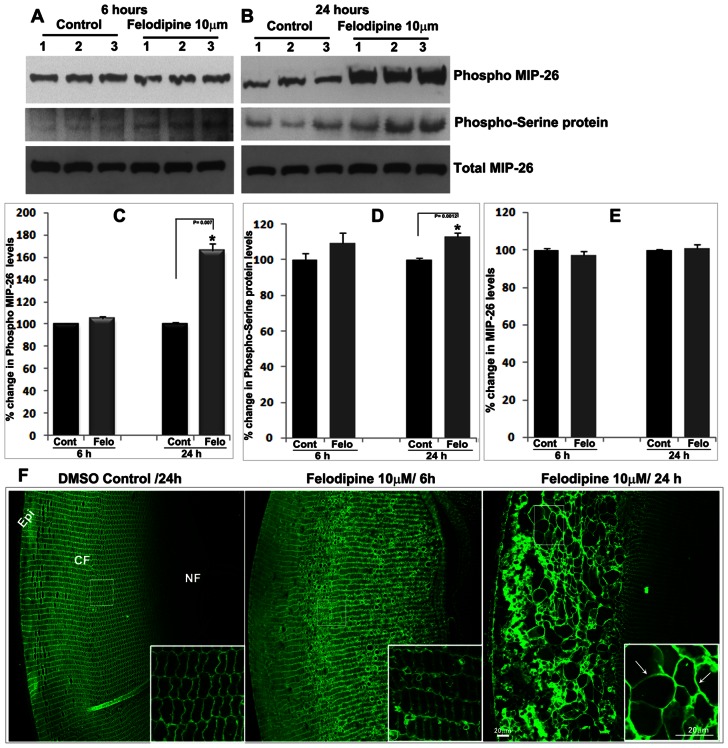
Inhibition of LTCCs by felodipine in ex-vivo organ cultured mouse lenses leads to increased Aquaporin-0 Ser235 phosphorylation. To determine the influence of LTCC inhibition on aquaporin-0 expression and phosphorylation, control lenses and lenses treated with felodipine (10 µM) for 6 (A) and 24 (B) hrs were examined for changes in the levels of total aquaporin-0 (MIP-26), Ser235-phosphorylated aquaporin-0 (Ser235 Phospho-MIP26) and serine phosphorylated proteins in membrane-enriched lens insoluble fractions, This analysis was performed by immunoblotting using the respective antibodies (see Methods) and subsequent quantification with Image J based densitometric analysis (Panels C, D and E). The levels of both Ser235 phosphorylated MIP-26 (aquaporin-0) and serine phosphorylated proteins (∼28 kDa) exhibited significant increases (* P<0.01; n = 4) in felodipine treated (24 hrs) specimens compared to control lenses. The levels of total MIP-26 were found to be unaltered by felodipine treatment. Total MIP-26 was used as a loading control for the phospho-MIP26 analysis. Samples derived from three independent lenses were depicted from both the felodipine and DMSO treated groups. Panel F depicts the distribution of MIP-26 (aquaporin-0) in equatorial sections from felodipine and DMSO treated lenses based on immunofluorescence analysis using a MIP-26 polyclonal antibody. Inserts in Panel F show fiber cell swelling in the felodipine treated specimens (indicated with arrows). Bar indicates magnification. Epi: Epithelium, CF: cortical fibers, NF: Nuclear fibers.

As described earlier, felodipine treated lenses exhibit decreases of lens weight (Fig. 2) and extensive fiber cell swelling (Fig. 3) indicating possible changes in water permeability and transport. Since water channel activity of aquaporin-0, which is the most abundant water channel protein in the lens fiber cell membrane, is known to be regulated by calcium and calmodulin in a phosphorylation (Ser235) dependent manner, [29], [33], [34], [35] we examined changes in aquaporin-0 serine235 phosphorylation in drug-treated lenses, using a ser235 phospho-specific polyclonal aquaporin-0 antibody. Aquaporin-0 ser235 phosphorylation which is regulated by protein kinase A and AKAP2 (A-kinase anchor protein-2) was recently demonstrated to be crucial for the water permeability activity of aquaporin-0. [29] The total aquaporin-0 protein levels in the membrane enriched insoluble lens fraction (P21) were found to be similar between felodipine treated and control specimens following 6 or 24 hrs of drug treatment (Fig. 5A, B and E). Additionally, despite extensive disorganization and swelling of fiber cells in drug-treated lenses (Fig. 5F, inserts), aquaporin-0 was found to exhibit a normal pattern of distribution to the fiber cell membrane, as determined by immunofluorescence staining (Fig. 5F, arrows in insert). However, significant increases were evident in the level of ser235 phosphorylated aquaporin-0 in lenses treated with felodipine for 24 hrs (Fig. 5B and C). We also measured changes in serine-phosphorylated proteins in membrane-enriched fractions from felodipine-treated lenses using a monoclonal antibody specific for the serine-phosphorylated residue in proteins. The same ∼28 kDa molecular mass protein which exhibited intense reactivity with ser235 phospho-specific aquaporin-0 antibody also cross reacted with the phosphoserine antibody, exhibiting elevated levels in felodipine treated lenses compared to control lenses (Fig. 5A, B and D). These two observations collectively reveal that inhibition of LTCCs in mouse lenses increases aquaporin-0 ser235 phosphorylation.

**Figure 6:**
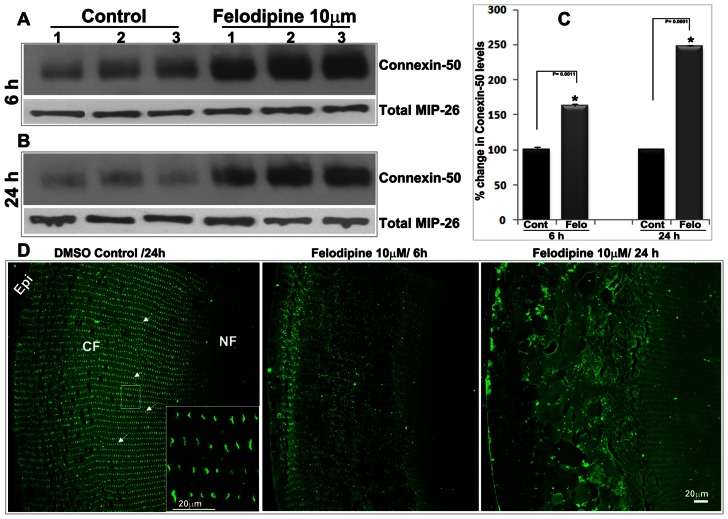
Increased levels of connexin-50 in LTCC inhibited ex-vivo organ cultured mouse lenses. To evaluate whether inhibition of LTCCs influences connexin-based gap junction activity, membrane enriched fractions from felodipine and DMSO treated control mouse lenses were examined for changes in connexin-50 protein levels by immunoblot analysis. The levels of connexin-50 were increased significantly in the felodipine treated lenses following 6 hrs (A) and 24 hrs (B) of drug treatment compared to control lenses. Total MIP-26 was used as a loading control for the connexin-50 immunoblots. Lanes 1 to 3 in panel A represent individual specimens from both groups. Panel C shows quantitative changes in connexin-50 in felodipine treated and control specimens, based on densitometric analysis of an n = 4 independent samples. Panel D shows disorganization of connexin-50 distribution in the felodipine treated lens cortical fibers (equatorial sections) relative to control specimens in which connexin-50 distribution is well organized and presents a clustered localization at the fiber cell long arm (see arrows & insert), based on immunofluorescence analysis using connexin-50 polyclonal antibody. Bar indicates magnification. Epi: Epithelium, CF: Cortical fibers, NF: Nuclear fibers.

In addition to aquaporin-0, we examined possible changes in the distribution and levels of connexins in felodipine-treated lenses, since the gap junction activity of these proteins, which are abundantly expressed in fiber cells, is essential for lens transparency. [5], [48] As shown in Fig. 6, connexin-50 is distributed in a precisely organized manner, exhibiting a clustered or patchy localization (Fig. 6D, arrows and insert) at the center of the long arm of hexagonal fiber cells based on immunofluorescence confocal imaging of sections from the lens equatorial plane. On the other hand, felodipine treated lenses exhibit a complete disorganization of connexin-50 distribution in the cortical fibers, with no disruptions noted in nuclear fibers (Fig. 6D). Intriguingly, the levels of connexin-50 were found to exhibit a robust and significant increase in membrane-enriched lens fractions from felodipine treated lenses relative to control lenses (Fig. 6 A, B and C), starting from 6 hrs of treatment with felodipine. Total aquaporin-0 was immunoblotted as a loading control in experiments designed to assess changes in levels of connexin-50. Connexin-46 protein levels and distribution were not examined in this study.

**Figure 7:**
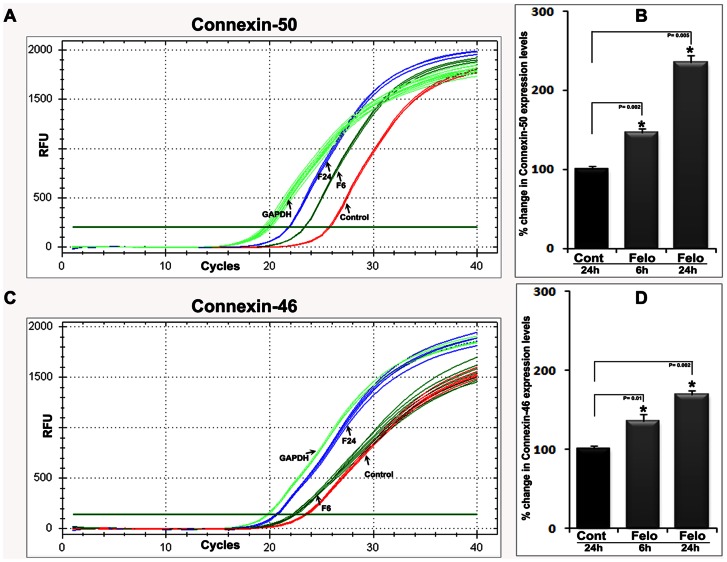
Upregulation of Connexin-50 and -46 gene expression in the felodipine treated mouse lenses. q-RT-PCR was performed to determine whether there is any difference in the transcriptional regulation of connexin gene expression in felodipine treated mouse lenses. For this, total RNA extracted from the felodipine treated (6 and 24 hrs) and control (DMSO treated) lenses was reverse-transcribed and used for q-PCR analyses. Real-time quantification of expression of connexin-50 and -46 genes was normalized to the cycle value (*Cycles*) of GAPDH. *Left panels*: plots of log fluorescence units versus cycle number. *Right panels*: relative percent fold change in connexin-50 and -46 gene expression with felodipine treatment in ex-vivo mouse lens. Fold changes were calculated based on the mean values from triplicate analyses of individual samples. F6 and F24 represent the 6 and 24 hr felodipine treated specimens, respectively.

Additionally, to determine whether the noted increase in the levels of connexin-50 was due to increased gene expression, felodipine treated lenses were examined for changes in the levels of connexin-50 and 46 transcripts by q-RT-PCR analysis (Fig. 7). This analysis revealed a significant increase in the levels of both connexin-50 and 46 transcripts in felodipine treated lenses relative to control lenses (Fig. 7A–D). Figures 7A and C depict the q-RT-PCR output traces for both connexin-50 and 46, respectively, along with the GAPDH control as indicated with arrows. Figures 7B and D show quantitative changes in the expression level of connexin-50 and 46 genes, respectively, in the felodipine treated lenses as compared to control lenses, based on results from three independent analyses. Expression levels of the GAPDH (Glyceraldehyde 3-phosphate dehydrogenase) transcript were used to normalize the cDNA concentration of control and drug treated lenses in q-RT-PCR analyses (Fig. 7A and C).

**Figure 8:**
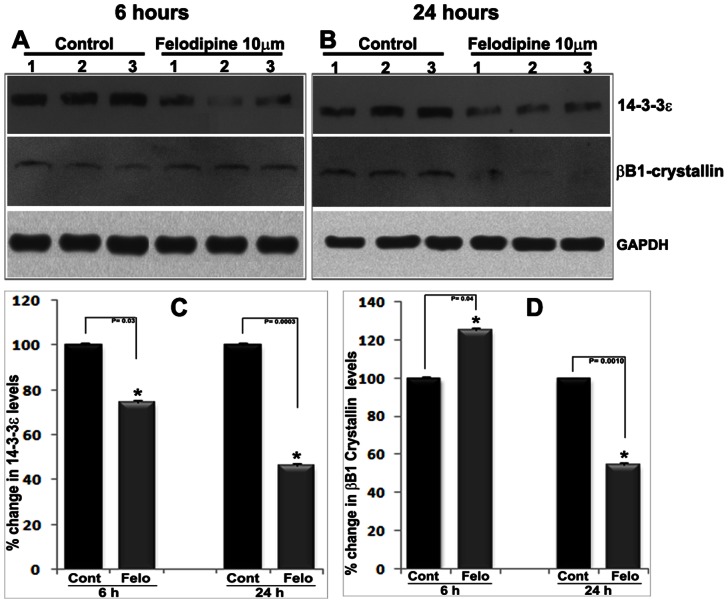
Decreased levels of 14-3-3ε, a phospho-serine/threonine protein binding protein in LTCC inhibited mouse lenses. Analysis of mouse lenses treated with felodipine (10 µM) for 6 hrs (A) and 24 (B) hrs revealed a significant decrease in the levels of 14-3-3ε compared to DMSO treated control lenses, as assessed by immunoblot analysis of lens soluble protein fractions in conjunction with densitometric analysis (Panel C; n = 4). The levels of βB1-crystallin exhibited modest but significant increases in the same samples after a 6 hr treatment with felodipine (A). After 24 hrs of drug treatment (B), however, a significant decrease was noted in the levels of βB1-crystallin in felodipine treated lenses relative to control lenses, based on immunoblot (A & B) and densitometric analyses (D; n = 4). GAPDH staining was used as a loading control for both the analyses.

As described earlier, felodipine treated lenses revealed changes in the profile of different proteins including 14-3-3ε and β-B1-crystallin, based on SDS-PAGE analysis followed by mass spectrometry -based identification (Fig. 4C; Table 1)). Since 14-3-3ε and other isoforms of this protein are known to interact with and regulate the membrane organization and activity of various channel proteins, [49], [50], [51], [52], [53], [54] we evaluated the effects of felodipine on the levels of this protein in drug-treated mouse lenses. The levels of 14-3-3ε protein decreased progressively and significantly in felodipine-treated lenses starting from 6 hrs onwards, based on immunoblot analysis of soluble lens fractions (Fig. 8A, B & C). On the other hand, the levels of β-B1-crystalin, which is a calcium binding crystallin, [37] exhibited modest increases in the 6 hrs felodipine-treated lenses (soluble fraction) (Fig. 8A & D), but at 24 hrs following drug treatment, the levels of this protein decreased significantly compared to the levels in control lenses (Fig. 8B and D). GAPDH was used as a loading control for these analyses.

### Impaired Myosin Light Chain Phosphorylation Precedes Lens Opacity Induced by LTCC Inhibition

**Figure 9:**
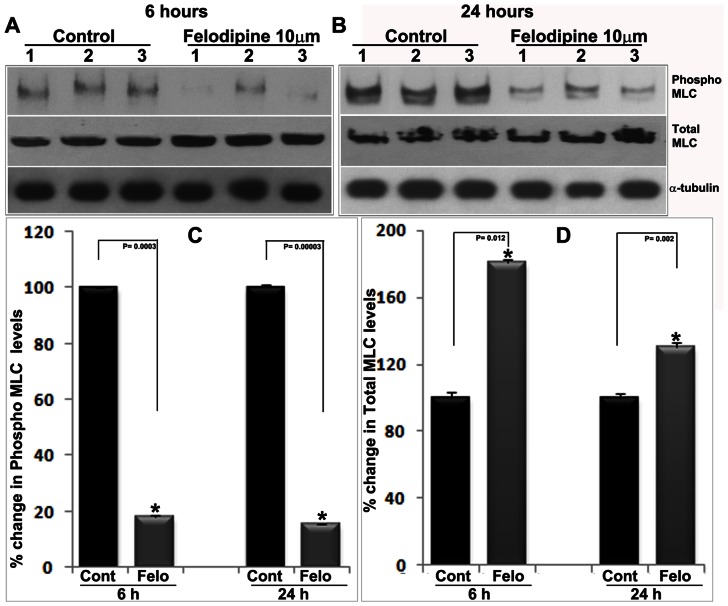
Inhibition of LTCCs in ex-vivo organ cultured mouse lenses by felodipine impairs myosin light chain phosphorylation. To determine the effects of inhibiting LTCC activity by felodipine (10 µM) on MLC phosphorylation and total MLC levels in the lens, felodipine and DMSO (control) treated mouse lenses were analyzed for changes in the levels of phospho-MLC and total MLC by immunoblot analysis. The levels of phospho-MLC exhibited marked and significant decreases in felodipine treated lenses after 6 hrs (A) and 24 hrs (B) of drug exposure, relative to control lenses. Interestingly, the same samples also exhibited significant increases in the level of total MLC following either a 6 hr or a 24 hr exposure to felodipine, as compared to the corresponding control samples (A & B). Panels C and D show quantitative changes in the levels of phospho-MLC and total MLC respectively in felodipine treated lenses based on densitometric analysis of 4 independent specimens. Lanes 1 to 3 represent three independent samples from both control and drug treated groups.

Myosin light chain (MLC) which is a regulatory subunit of myosin II and whose phosphorylation is regulated by calcium/calmodulin-dependent MLC kinase, is critical for actomyosin-based contractile and mechanical activity. [55] We evaluated MLC phosphorylation to determine whether impairment of LTCC activity by felodipine indeed influences MLC phosphorylation in lens tissue. This was done by immunoblotting analysis using a phospho-specific MLC antibody (Fig. 9). In lenses treated with 10 µM felodipine for either 6 or 24 hrs, there was a significant (>80%) decrease in MLC phosphorylation compared to control lenses (Fig. 9 A, B and C). In contrast, the levels of total MLC protein were significantly elevated in the same fractions from the felodipine treated lenses, based on immunoblotting analysis and as compared to control lenses (Fig. 8 A, B and D). This response was found to be much robust in lenses treated for 6 hrs with felodipine as compared to lenses treated with the drug for 24 hrs (Fig. 9D). For both phospho-MLC and total MLC analyses, α-tubulin was immunoblotted as a loading control.

### LTTC Inhibition in Mouse Lens Epithelial Cells Disrupts Actin Cytoskeletal Organization and Decreases Intracellular Calcium and MLC Phosphorylation

**Figure 10:**
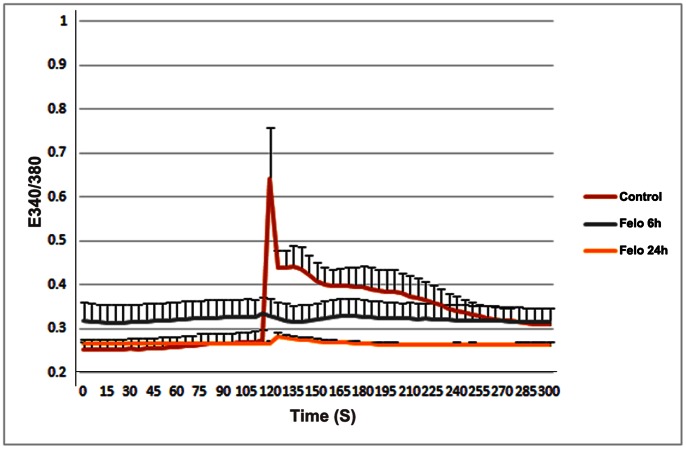
Decreased intracellular calcium in felodipine treated mouse lens primary epithelial cells. To confirm the effects of felodipine on intracellular calcium levels, lens epithelial cells derived from P26 mouse lenses were cultured in glass bottom plastic culture chambers and treated with felodipine (10 µM) as described in the Methods section, for 6 or 24 hrs. The drug treated and control cells loaded with FURA-2/AM (4 µM) for 30 min and were rinsed with Hank’s balanced salt solution prior to acquisition of baseline FURA-2AM fluorescence measurements in several individual cells using digital imaging fluorescence microscope at excitation 340/380 nm for 2 min. Following this, while the recoding was ongoing, thapsigargin (1 µM) was added to the cells and FURA-2/AM fluroscence recording continued for an additional 3 min to detect the thapsigargin-mediated depletion of intracellular calcium stores. Based on these analyses and as shown in the figure, only control cells revealed a significant increase in calcium-dependent FURA-2/AM signal from their respective baseline signal. On the other hand cells treated with felodipine for either 6 or and 24 hr did not exhibit any significant increase in calcium signal from their respective baseline values, confirming a marked decrease in total intracellular calcium in the felodipine treated lens epithelial cells compared to the controls. Values represent mean ± Standard deviation based on several independent cell recordings.

To obtain further insight into the influence of LTCC activity on the regulation of MLC phosphorylation, actin cytoskeletal organization and intracellular calcium, lens primary epithelial cells, derived from P26 C57BL/6J mice were treated with felodipine (10 µM) for either 6 or 24 hrs in serum free medium. The FURA-2/AM-loaded lens epithelial cells were evaluated by digital imaging microscopy for baseline fluorescence at 340/380 nm excitation in the presence and absence of thapsigargin. As shown in Fig. 10, while control cells exhibited a robust and sharp spike/elevation in the calcium-dependent FURA-2/AM fluorescence with addition of thapsigargin, the felodipine- treated lens epithelial cells from both 6 and 24 hrs treatments failed to show any significant calcium signaling in response to thapsigargin, indicating a significant reduction in total intracellular calcium in the LTCC-inhibited cells. These conclusions were based on recordings with several individual cells.

Felodipine treated lens epithelial cells were examined for changes in cell viability and cytotoxicity using in vivo fluorescein diacetate hydrolysis and propidium iodide labeling. As shown in the supplemental figure (Figure S1), the felodipine treated lens epithelial cells were found to be viable based on in vivo imaging of fluorescein diacetate hydrolysis –dependent green fluorescence and lack of propidium iodide staining, similar to what is noted for control cells. These data confirm lack of drug-induced cytotoxic effects in lens epithelial cells (Figure S1) and are consistent with the noted lack of apoptosis in the felodipine treated lenses described earlier (Fig. 3D).

**Figure 11:**
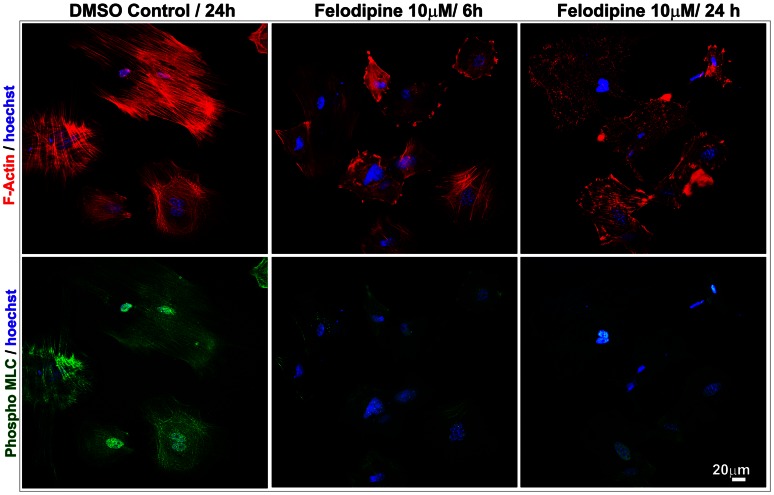
LTCC inhibition by felodipine decreases actin stress fibers and MCL phosphorylation in mouse lens epithelial cells. Mouse lens primary epithelial cells cultured on gelatin-coated glass cover slips and treated for 6 or 24 hrs with felodipine (10 µM) in the absence of serum revealed a dramatic decrease in actin stress fibers (red) stained with rhodamine phalloidin and phospho-MLC (green) stained with phospho-MLC antibody in conjunction with FITC-conjugated secondary antibody compared to the DMSO treated control cells. Blue stain represents cell nuclei labeled with Hoechst stain. The images are representative of triplicate analyses. Scale bar indicates magnification.

Importantly, the felodipine treated lens epithelial cells revealed a dramatic decrease in actin stress fibers and MLC phosphorylation as assessed by rhodamine phalloidin staining (red) and phospho-MLC antibody immunofluorescence staining (green), respectively (Fig. 11). While control cells exhibit strong actin stress fibers (red staining) extending from one end to another end and distributed throughout the cell body, barely any actin stress fibers were evident in the felodipine treated cells, indicating drug-induced actin depolymerization. Consistent with the changes observed in actin stress fibers, drug treated lens epithelial cells exhibited very little staining for phospho-MLC as compared with the intense and filamentous staining distributing along the actin stress fibers in control cells (Fig. 11). The merging of actin stress fiber staining with phospho-MLC staining confirms perfect colocalization of these two proteins (data not shown). The blue staining depicts cell nuclei labeled with Hoechst. Representative images of individual cells derived from triplicate analyses are shown.

## Discussion

This study reveals the expression and distribution profile of Ca (V) 1.2 and 1.3 voltage-gated LTCCs in mouse lens epithelium and fiber cells, and demonstrates that pharmacological inhibition of these LTCCs impairs lens transparency, together with increased aquaporin-0 serine- 235 phosphorylation which is known to directly modulate the water permeability activity of aquaporin-0. Treatment of mouse lenses with LTCC inhibitor did not cause any detectable cytotoxicity, but was found to upregulate gene expression of connexin-50 and -46 which are key fiber cell gap junction proteins involved in regulation of lens microcirculation. Additionally, impairment of LTCC activity in the mouse lens leads to decreased phosphorylation of MLC, which is a regulatory subunit of myosin II required for maintenance of lens cytoarchitecture and mechanical properties. Cultured mouse lens epithelial cells treated with the LTCC inhibitor felodipine also exhibit decreased actin stress fibers, MLC phosphorylation, and decreased intracellular calcium, in the absence of any cytotoxic responses. These observations collectively uncover a critical role for LTCCs in homeostasis of lens transparency and architecture.

Intracellular calcium is an essential and versatile intracellular messenger and regulates variety of cellular functions. [56], [57] Therefore, a tightly regulated calcium influx is required for homeostasis of cell and organ function. Some of our earlier intriguing observations in the ocular lens including identification of various scaffolding proteins involved in ion channel protein membrane clustering, [7] force-induced changes in MLC phosphorylation (unpublished data) and high levels of phosphorylated MLC in lens fibers [42] along with the known rapid changes in lens shape during accommodation, motivated us to examine whether LTCCs which regulate intracellular calcium, might play a significant role in lens transparency and structural integrity. The role of LTCCs in neuronal and cardiac muscle excitability, contraction coupling, various biochemical responses and gene expression has been well understood and demonstrated to have a vital role in physiology and function of various organs. [45], [58], [59] LTCCs are multimeric complexes consisting of pore forming α1 subunit and auxiliary β, α2δ and γ subunits. The α1 subunit serves as the main functional component of the channel complex. There are 4 different isoforms of α1 (Ca V 1.1, 1.2, 1.3 and 1.4) encoded by 4 different genes. [45], [60], [61] The activity of these channel proteins is regulated by various extracellular cues and intracellular signaling mechanisms. Our data based on using multiple approaches revealed the expression and distribution of Ca (V) 1.2 and 1.3 in both lens epithelium and cortical fiber cells. Interestingly, the distribution of these channel proteins in fiber cells was discretely and intensely localized to the short arms of the hexagonal lens fibers. Additionally, relative to Ca (V) 1.3, which is distributed uniformly to the short arm, Ca (V) 1.2 was found to be clustered at the short arm. The reason for these differences in the distribution pattern of these two LTCCs within lens fiber cells is not clear at present. However, Ca (V) 1.2 and 1.3 are known to influence different cellular responses despite their similar activity as it relates to regulation of intracellular calcium. [45], [60], [61].

The role of LTCCs in maintenance of normal lens transparency and structural integrity is not understood. The data from this study demonstrate that LTCC activity is critical for normal lens transparency based on felodipine and nifedipine induced opacity in ex-vivo lenses. The subcapsular and cortical opacity induced by these agents is correlated with the intense distribution of LTCCs in the cortical fibers. Additionally, this body of work regarding the importance of LTCC activity for normal lens transparency further supports the earlier reported clinical observation of nifedipine use posing an increased risk for cataract formation in human subjects. [62], [63] Intriguingly, the felodipine and nifedipine treated mouse lenses which exhibited cortical opacity, also revealed decreases in lens weight without any noticeable cytotoxic response. Although there was an increase in protein leakage from the LTCC inhibited lenses, the amount of protein leakage from these lenses was not very large as compared to the protein losses from control lenses. Additionally, the control lenses did not exhibit any opacification despite the noted protein leakage during lens incubation. Therefore, it is possible that in addition to protein leakage, altered microcirculation and water permeability could be partly responsible for the observed decreases in lens weight. The basis for this latter speculation is the fact that histological changes including disorganization and swelling of cortical fibers noted in the LTCC inhibited lenses appeared very similar to the changes reported in the mouse lenses with altered aquaporin-0 water channel activity in ex vivo. [29].

In contrast to our data on LTCC inhibitor induced opacification in ex-vivo normal lenses, other LTCC inhibitors including verapamil and nifedipine have been reported to delay formation of diabetic and radiation-induced cataracts in rodents. [38], [39], [40], [41] Diabetic and radiation-induced cataract lenses have been reported to contain elevated levels of intracellular calcium which is expected to activate calpain and caspases and cause protein degradation and cell death, respectively. [38], [41], [64] Additionally, diabetic lenses are reported to have elevated levels of sugar alcohols such as sorbitol and xylitol which can lead to osmotic imbalance and lens opacification. [65] Since inhibition of LTCCs is expected to decrease intracellular calcium in lens epithelial cells as documented in our studies (Fig. 10) and those from other laboratories [66], LTCC inhibitors are presumed to have a protective response in delaying lens opacification in diabetic and radiation-exposed rodents as noted in several published studies. [38], [39], [40], [41] Additionally, elevated levels of polyols are thought to cause osmotic imbalance leading to water accumulation in extracellular spaces in the diabetic lenses. [67] It is plausible that the LTCC inhibitor mediated increase in Ser235 phosphorylation of aquaporin-0 noted in this study with expected subsequent enhancement in aquaporin-0 water permeability [29] might mediate a protective effect towards delaying cataract formation in diabetic lenses by increased influx of water from the extracellular environment into cell interior and subsequently coupling to the lens microcirculation system. However, in contrast to sugar and radiation-induced cataracts, inhibition of LTCCs in normal lenses, per se appears to cause a variety of biochemical and metabolic insults due to decreased intracellular calcium including transcriptional changes, changes in cytoskeletal organization and integrity, deregulated water channel, gap junction and myosin II activities. Collectively these effects appear to impair lens transparency under normal conditions.

Our studies revealed increased levels of connexin-50 in the membrane-enriched fraction of lenses treated with felodipine relative to control lenses, with the increases preceding any noticeable lens opacification. Interestingly, the increased levels of connexin-50 in the felodipine treated lenses appear to be the result of transcriptional upregulation based on the q-RT-PCR results (Fig. 7). Additionally, as shown in this study, the expression of connexin-46 gene was also found to be upregulated under LTCCs inhibition indicating a close regulatory interaction between the homeostasis of intracellular calcium and transcriptional control of connexin gene expression in the lens tissue. On the other hand, while the total aquaporin-0 levels were found to be similar between the felodipine treated versus control lenses, the levels of ser235 phosphorylated aquaporin-0 were elevated significantly in felodipine treated lenses. It has been reported that Aquaporin-0 ser235 phosphorylation is partly regulated by calcium/calmodulin and PKA. [29], [34] Calmodulin has been shown to bind to the C-terminal domain of aquaporin-0 near ser235 in a calcium dependent manner [28], [33], [34], [68] and mask this site for ser235 phosphorylation by PKA. [29] This blockage of PKA-regulated ser235 phosphorylation of aquaporin-0 by calcium/calmodulin is thought to impair aquaporin-0 water permeability activity by closing the channel pore. [29] On the other hand, the absence or decreased levels of intracellular calcium impairs the complex formation of calcium/calmodulin with aquaporin-0, facilitating the PKA/AKAP2 mediated ser235 phosphorylation and activation of the pore activity of aquaporin-0 for water, thereby increasing influx of water. [29] Therefore the increase in aquaporin-0 ser235 phosphorylation observed in our studies upon LTCC inhibition, when taken together with increased fiber cell swelling in cortical fibers, strongly suggests that the LTCC regulated calcium influx directly or indirectly influences aquaporin-0 water channel activity and fiber cell water permeability. Interestingly, aquaporin-0 has been shown to interact directly connexin-50 and this interaction has been shown to enhance connexin-50 gap junction coupling. [69], [70] Therefore, it is conceivable that there may be also some possible relationship between aquaporin-0 phosphorylation and connexin-50 expression in the LTCC inhibited lenses.

It is also noteworthy that 14-3-3ε, a serine/threonine phospho-protein binding protein which is known to regulate activity and biogenesis of LTCC and other channel proteins, [49], [50], [51], [52], [53], [54] was found to be decreased in the felodipine treated lenses before any significant lens opacification had occurred. Interestingly, the protein levels of this broad family of regulatory proteins are reported to be decreased upon inhibition of Na-K ATPase activity in the lens, indicating their close functional association with transport or channel protein activity in lens. [71], [72] Additionally, PKCγ interaction with 14-3-3ε has been shown to influence connexin-43 gap junction activity in lens epithelium. [73].

Our observation of a marked decrease in MLC phosphorylation in LTCC inhibited lenses together with significantly increased levels of total MLC reveals the importance of LTCC regulated intracellular calcium in regulation of myosin II activity in lens. This conclusion was also partly supported by the data obtained from lens epithelial cells in which inhibition of LTCCs was associated with decreased intracellular calcium, and decreased actin stress fibers and MLC phosphorylation (Fig. 10 and 11). It is possible that the elevated levels of total MLC in the felodipine treated lenses could be an adaptive response to the severely compromised activity of MLC. Additionally, in support of the importance of myosin II activity for lens transparency and architecture, in our previous study we found that inhibition of MLC kinase which is a calcium/calmodulin-dependent kinase, leads to lens opacification. [42] Although the precise role of the robust increases in levels of phosphorylated MLC in differentiating lens fibers reported earlier is yet to be understood, [42] changes in lens shape induced by physical force caused decreases in MLC phosphorylation, suggesting an involvement of force sensitive ion channels in regulation of intracellular calcium (our unpublished data). One of the unique characteristics of lens is its rapid deformability during accommodation. Whether lens deformability and the associated biomechanical and shape changes in fiber cell is partly influenced by intracellular calcium and contractile properties are not known. The role of LTCC activity in cardiac contractile coupling is recognized to be important for heart function. [58], [61], [74] Therefore, additional studies are necessary to explore and delineate the role of LTCC activity in lens contractile and tensile properties during lens accommodation using primate lenses. In addition, it is well recognized that myosin II activity is essential for lens function based on cataract formation in both human and mouse models with mutation in nonmuscle myosin II-A (MYH9). [75], [76] In future studies, we plan to determine whether inhibition of LTTC activity influences lens stiffness to unravel the significance of LTCCs in maintaining the lens mechanical properties and function. Finally, in this initial study we demonstrate the importance of LTCC activity for maintenance of lens transparency. The precise role, significance and mechanistic understanding of the noted changes in aquaporin-0 phosphorylation, expression of connexins and levels of 14-3-3ε which occur upon inhibition of LTCC, however, require additional in vivo and gene targeted studies.

## Methods

### Ethics Statement

#### Animals

This study was carried out in strict accordance with the recommendations of the Guide for the Care and Use of Laboratory Animals of the National Institutes of Health and the Association for Research in Vision and Ophthalmology. The protocol was approved by the Committee for Ethics of Animal Experiments at the Duke University School of Medicine (Protocol Number: A007-11-01).

### Reagents

Felodipine (C. No. F9677), Nifedipine (C. No. N7634), α*-*Tubulin mouse monoclonal antibody (C. No.T9026), Wheat Germ Lectin (TRITC tagged, C. No. L5266), Fluorescein diacetate (Cat. No. 201642), Phalloidin–Tetramethyl rhodamine B isothiocyanate (C. No. P1951), Propidium iodide (C. No. P4170) and Thapsigargin (C. No. T9033) were procured from Sigma-Aldrich, St. Louis, MO. Dulbecco’s Modified Eagle growth Medium (Low Glucose, 298 mOsm) and Penicillin-Streptomycin-Glutamine, were purchased from Life Technologies, Grand Island, NY. L-type calcium channel mouse monoclonal antibodies against CaV1.2 (C. No. 75-053) and CaV1.3 (C. No. 75-080) were procured from Antibodies Incorporated Davis, CA. Phospho-Myosin Light Chain 2 (C. No. 36745) and total myosin light chain (C. No. 3672S) rabbit polyclonal antibodies were from Cell Signaling Technology, Inc. Danver, MA. 14-3-3ε (C. No. sc-23957 ) mouse monoclonal antibody was from Santa Cruz Biotechnology, Inc, Santa Cruz, CA. Connexin-50 rabbit polyclonal antibody (C. No.CX50-A) was purchased from Alpha Diagnostic International, San Antonio TX. Anti-Phospho-Serine antibody (C. No. ab6639) was from Abcam, Cambridge, MA. βB1-Crystallin and Aquaporin-0 rabbit polyclonal antibodies were generously provided by Sam Zigler, Johns Hopkins University School of Medicine and Joe Horwitz, Jules Stain Eye Institute, UCLA, respectively. Serine-235 phosphorylated Aquaporin-0 rabbit polyclonal antibody was a generous gift from Kevin Schey, Vanderbilt University School of Medicine. Mouse monoclonal antibody to GAPDH (C. No. IMG-5019A-2) was from Imagenex, San Diego CA. Medical background Sniper reducing solution was from Biocare (C. No. BS966L), Concord, CA, Alexa fluor 488 and Alexa fluor 568 conjugated secondary antibodies, Hoechst 33258 and FURA-2/AM (C. Nos. A-11008, A-11031, H3569 and F-120, respectively) were from Invitrogen, Carlsbad, CA. Hank’s Balanced Salt Solution (C. No. 14025-092) was from Invitrogen, Grand Island, NY). Glass bottom microwell cell culture chambers were from MatTek (C. No. P35G-1.5-14-C), Ashland, MA. ApopTag® Plus Fluorescein *In Situ* Apoptosis Detection kit (C. No. S7111) was from Chemicon Int, Temecula, CA. VectaMount (C. No. H-5000) was from Vector Laboratories, Burlingame, CA. PCR primer sets were ordered from Integrated DNA Technologies, Coralville, Iowa. RNeasy Micro kit (C. No. 74004) was from Qiagen, Valencia, CA. Advantage RT for PCR- kit (C. No. 639506) and Advantage® 2 PCR Kit, (C. No 639207) were from BD Biosciences Clontech, Palo Alto, CA. Bio-Rad protein assay reagent (C. No. 500-0006), Criterion XT precast gels (4–20%, C. No.345-0032) and iQ™ SYBR® Green supermix (C. No. 170-8880) were purchased from Bio-Rad, Hercules. GelCode® Blue staining reagent (C. No. 24590) was from Thermo Fisher Scientific Inc. Rockford, IL.

### Mice

C57BL/6J strain mice obtained from Taconic Laboratories (Hudson, NY) were bred in-house at the Duke University Medical Center Vivarium facility and used in this study. Lenses obtained from weaned mice (male and female animals aged between P21 to P26 days) were used in the organ culture studies. Animals were euthanized using intraperitoneal injections of Euthasol.

### Lens Organ Cultures

Freshly dissected lenses from 3-week-old mice were incubated at 37°C under 5% CO_2_ in Dulbecco’s Modified Eagle Medium (Low Glucose DMEM, 298 mOsm) containing penicillin (100 U/ml) and streptomycin (100 mg/ml). Lenses were stabilized in the culture media for 16 hrs and pre-screened to rule out any potential dissection-associated damages by assessing for protein leakage into the media as described earlier. [42] Pre-screened clear lenses were cultured in control media or media containing felodipine or nifedipine dissolved in dimethyl sulfoxide (DMSO, at a final concentration of 0.025%). Lenses treated with DMSO (0.025%) alone served as controls for drug treatments. Media were changed every day for both the control (DMSO treated) and drug-treated lenses, lens transparency was monitored and graded daily using a dissecting light microscope and images were recorded under dark illumination using Canon digital camera (Zeiss AXio Cam ERc 5 s). Felodipine (10 µM) treated lenses were harvested at 6, 24 and 48 hrs intervals, while nifedipine (25 µM) treated lenses were harvested after 48 hrs. DMSO treated control lenses were harvested at the respective time intervals mentioned above. Following drug treatment, lenses were fixed and stored at the appropriate conditions for analysis of histological changes by hematoxilin and Eosin (H&E) staining, immunohistochemistry, total protein profiles and immunoblotting.

### Mouse Lens Epithelial Cultures

Mouse lens primary epithelial cells were isolated by collagenase IV digestion of lens capsules derived from P26 C57BL/6J strain mice, as described earlier by us. [77] Third- and fourth-passage cells were cultured at 37°C under 5% CO_2_, in Dulbecco’s modified Eagle’s medium (DMEM) containing 10% fetal bovine serum (FBS) and penicillin (100 U/mL)-streptomycin (100 µg/mL)-glutamine (292 µg/mL).

### Cell Viability and Cytotoxicity

To evaluate the effects of felodipine on the viability of mouse lens epithelial cells, cells were grown on gelatin-coated 12 well plastic plates in the presence of DMEM and FBS as described above. After treatment with 10 µM felodipine for 6 or 24 hrs in the absence of FBS, cells were rinsed twice with DMEM and then treated with fluorescein diacetate (2 µg/mL ) and propidium iodide (0.6 µg/mL) for 3 minutes. Following this, viable and dead/damaged cells, which stain green and red, respectively, were viewed and imaged using a Zeiss Observer.D1 fluorescence microscope.

### Intracellular Calcium Assay

Changes in the levels of intracellular calcium were determined using FURA-2/AM in mouse lens epithelial cells treated with felodipine, as previously described. [78] Briefly, mouse lens epithelial cells were seeded in glass-bottomed 35-mm Petri dishes and were allowed to grow for three days in DMEM with FBS (10%). The cells were then rinsed with DMEM (no FBS) and treated with felodipine (10 µM) for 6 to 24 hrs in the absence of FBS. Following this, the cells were incubated with FURA-2/AM at a final concentration of 4 µM for 30 min at 37°C in the dark. The cells were then washed twice with Hanks’ balanced salt solution followed by recording base-line measurements of relative intracellular concentration in control and felodipine- treated cells using digital imaging microscopy at 340/380 nm excitation. After obtaining base-line measurements, thapsigargin (1 µM) was added to the culture media, and measurements were continued for over 3 minutes at 5 seconds interval. Intracellular calcium levels in control and drug treated cells in the presence and absence of thapsigargin was expressed as the ratio of emitted fluorescence of cytoplasmic FURA-2/AM at 340/380-nm light excitations. We collected data from 5–10 cells per high power microscope field. The data were collected and analyzed with SimplePCI 6 (Hamamatsu Corp., Serwickley, PA).

### RT-PCR

To determine the expression profile of genes encoding L-type Calcium channels and their auxiliary subunits in mouse lens tissue, we performed RT-PCR analyses using total RNA extracted from the P2 and P21-day-old mouse (C57BL/6 strain) lenses using RNeasy Micro kit as we described earlier. [7] The Advantage RT-for-PCR Kit was used to synthesize first-strand cDNA from total RNA isolates, followed by amplification of cDNA pools for the genes of interest, using an Advantage® 2 PCR Kit. Minus RT (reverse transcriptase) controls were included in the RT-PCR reactions. The mouse-specific forward and reverse oligonucleotide primer sets were used in PCR reactions to amplify cDNA species corresponding to various LTCC subunits (Table 2): The PCR gene products were extracted and sequenced to confirm identity.

**Table 2:** The list of Oligonucleotide Primer sets used in the RT-PCR and q-RT-PCR reactions.

Ca (V) 1.1	CTCCGCTATGATGTCACTCTTC/GACGACATACCACACCTGATAC
Ca (V) 1.2	ACAGCCAATAAAGCCCTCCTGGCCC/GGGAGGCAATGGAGCGCACTGAGTT
Ca (V) 1.3	T TGCTGTGAGGACGACAGCTCTCCCA/TAGGCCTGCAACGGCCATGATCTGC
Ca (V) 1.4	CGCAATGGCTGGAACCTGCTCGACT/GTGTGCATGAACGCCCAGAGCCAGA
α2δ	GAGTGAGCCAGGCAGCCAACGGATT/CGCTGCCAAACACTTGCCACAGCAG
β2	TCAGATACAGCGCAGCCCAGGAGGA/CAGCACCACTGGTCGCATGGAAGGT
Connexin-50	GAGAATGTCTGCTACGATGAGG/AGGATGCGGAAACCATACAG
Connexin-46	CATCGGGTTCCCACCTTATT/TACCAAGGCACTCTCCTGT
GAPDH	CCGAGCTGAGCATAGACATT/TCCACCACCCTGTTGCTGTA

### q-RT-PCR

Real-time quantification of connexin-50 and -46 gene transcripts in felodipine treated and control mouse lenses was performed using a Prism 7700 Sequence Detection System (Applied Biosystem, Inc), as previously described by us. [77] Briefly, first strand cDNA pools from the drug treated and control lenses were normalized relative to the housekeeping gene *GAPDH*. PCR reactions were done in triplicate using PCR Master Mix as described earlier. [77] The sequences of oligonucleotide primer sets of connexin-50 and -46 are listed in Table 2. The fold difference in connexin-50 and connexin-46 gene expression between control and felodipine treated lenses was normalized to *GAPDH* and calculated by the comparative threshold (C_T_) method, as described by the manufacturer (Prism 7700 Sequence Detection System; Applied Biosystem, Inc.).

### Histological Analysis

The organ cultured lenses were fixed and stored in 10% buffered formalin at room temperature until further processing. The specimens were subsequently dehydrated and embedded in paraffin, and 5-micron thick sections were cut in equatorial plane and mounted on the charged glass slides. These sections were subjected to H & E staining. Images were captured using Zeiss Axioplan2 microscopy (Zeiss Inc. Thornwood, NY. USA).

### Analysis of Lens Protein Profile in Cytosolic and Membrane Enriched Fractions

Control and drug treated cultured mouse lenses were homogenized using a glass homogenizer and cold (4°C) hypotonic buffer containing 10 mM Tris buffer pH 7.4, 0.2 mM MgCl2, 5 mM N-ethylmaleimide, 2.0 mM Na_3_VO_4_, 10 mM NaF, 60 µM phenyl methyl sulfonyl fluoride (PMSF), 0.4 mM iodoacetamide, Protease inhibitor cocktail (complete, Mini, EDTA-free) and PhosSTOP Phosphatase Inhibitor Cocktail (one each/10 ml buffer), Roche (Basel, Switzerland)). Homogenates were centrifuged at 800×*g* for 10 min at 4°C, and supernatant fractions were centrifuged further at 100,000×g for one hr at 4°C. The 100,000×g supernatant was designated as the cytosolic fraction, while the insoluble pellets derived were re-suspended in hypotonic buffer, washed twice with same buffer and the final membrane-enriched protein pellets were suspended in urea sample buffer containing 8 M urea, 20 mM Tris, 23 mM glycine, 10 mM dithiothreitol (DTT), and saturated sucrose along with protease and phosphatase inhibitors. Protein concentrations were estimated in both the cytosolic and membrane-enriched fractions by the Bradford method using protein assay reagent. Protein samples were separated by gradient SDS-PAGE (4–20% Criterion XT precast gels) using 1× MOPs buffer (Invitrogen) and stained overnight with GelCode® Blue stain reagent. After destaining with Milli Q pure water, gels were imaged using a Fotodyne image system, and protein bands of interest were excised from the gels and trypsin digested for further characterization by mass spectrometry. Briefly, gel slices were subjected to in-gel tryptic digestion using the In-Gel Tryptic digestion kit (Pierce), as per manufacturer’s instructions. This digestion process included both reduction and alkylation of protein samples.

### Mass Spectrometry

Peptides obtained from in-gel-trypsin-digests where analyzed using a nanoAcquity UPLC system coupled to a Synapt G2 HDMS mass spectrometer (Waters Corp, Milford, MA). Peptides were separated on a 100 µm×100 mm column containing 1.7 µm C18 BEH particles (Waters), and using a 30-min gradient of 5 to 32% acetonitrile with 0.1% formic acid at a flow rate of 0.3 µl/min and a column temperature of 35°C. For each sample, we conducted a data-dependent analysis (DDA) using a 0.8 s MS scan followed by MS/MS acquisition of the top three ions with charge greater than one. MS/MS scans for each ion used an isolation window of ∼3 Da, a maximum of 2 s per precursor, and dynamic exclusion for 120 s within 1.2 Da. DDA data were converted to searchable files using ProteinLynx Global Server 2.4 (Waters Corporation) and searched against NCBI mouse database (February 2011) using Mascot server 2.2 with the following parameters: maximum one missed cleavage site, carbamidomethylation at Cys residues as fixed modification and Met oxidation, N-terminal acetylation, Asn, Gln deamidation as variable modifications. Precursor ion mass tolerance was set to 20 ppm, while fragment mass tolerance to 0.2 Da. Acceptance criteria for protein identification required identification of at least two peptides for each protein with a confidence interval percentage, (CI %) of over 99.9%, corresponding to a false discovery rate of 0.1%.

### Immunoblotting

To determine the presence of Ca (V) 1.2 and Ca (V) 1.3 proteins in mouse lens, lens membrane-enriched protein fractions were isolated as mentioned above from P21 mice. Similarly cytosolic and membrane-enriched protein fractions were prepared from control (DMSO incubated) and drug treated lenses. Equal amounts of proteins were resolved by SDS-PAGE using 5, 8 or 15% acrylamide containing gels, followed by electrophoretic transfer to nitrocellulose membranes. Membranes were blocked with 5% milk protein, in Tris buffer containing 0.1% Tween-20 (washing buffer) and then incubated overnight with appropriate monoclonal or polyclonal primary antibodies, at a 1∶1000 dilution, followed by the respective secondary antibodies as described earlier. [7] Immunoblots were then developed using enhanced chemiluminescence ECL prime reagents (Amersham Biosciences, Buckinghamshire, UK; C. No. RPN2232), and scanned using a FOTODYNE Gel Doc scanner equipped with TL100 software, densitometry analysis was carried out using ImageJ software.

### MLC Phosphorylation

Following harvest, control (DMSO treated) and drug treated lenses were incubated with 10% ice cold trichloroacetic acid (TCA) for 5 minutes, washed with several changes of ice cold water, followed by diethyl ether, air dried and stored at -80°C until further processing. For extraction of total protein, lens specimens were homogenized in 8 M urea buffer containing 20 mM Tris, 23 mM glycine, 10 mM dithiothreitol and saturated sucrose along with protease and phosphatase inhibitors. Lysate was centrifuged at 16,000 xg for 15 min, and protein content analyzed in the supernatants. Equal amounts of protein (100 µg) from the supernatants derived from control and drug treated samples were resolved by urea/glycerol-polyacrylamide gel electrophoresis and immunoblotted using anti-phospho-specific MLC rabbit polyclonal antibody, as we described earlier. [42] Immunoblots were developed using enhanced chemiluminescence ECL prime reagents. Total MLC levels were also determined in the same samples by immunoblot analysis using anti-MLC antibody.

### Immunofluorescence

Tissue sections derived from either paraffin embedding or cryofixation were used in immunofluorescence analyses. The paraffin embedded lens sections were de-paraffinized and immunostained as we described earlier. [7] Slides were viewed and photographed using a Nikon Eclipse 90i confocal laser-scanning microscope. For double labeling, appropriate polyclonal and monoclonal antibodies were mixed and incubated with the tissue specimens as described above in conjunction with Alexa Fluor 488 and 568 conjugated secondary antibodies and Hoechst 33258.

For Cryosection immunostaining, freshly dissected mouse lenses (p21-old) were fixed in 4% paraformaldehyde in phosphate-buffered saline (PBS) at 4°C, overnight. Fixed lens tissues were then transferred sequentially into 5% and 30% sucrose in PBS. Tissues embedded in optimal cutting temperature medium (OCT) were sectioned using a cryotome (Microme HM 550, Walldorf, Germany), equatorial or sagittal plane (5–10 micron thick) tissue sections were mounted on charged glass slides and stored at -80°C until further processing. Cryosections were immunostained as we described earlier using appropriate primary and secondary antibodies. [7].

Mouse lens epithelial cells grown on gelatin-coated glass coverslips were stained for F-actin and phospho-MLC using rhodamine phalloidin and phospho-MLC antibody, respectively as previously described. [77].

### TUNEL Assay

In situ terminal transferase dUTP nick end labeling (TUNEL) staining was performed using an ApopTag Plus Fluorescein kit to evaluate and compare apoptotic cell death in mouse lens sections derived from felodipine treated and control lenses as described previously. [79] Apoptotic cells were detected using a fluorescence microscope (Zeiss Axioplan-II). In the same specimens, cell nuclei were labeled with Hoechst stain. The DNase-1 treated lens sections were used as a positive control for this assay.

### Statistical Analysis

Where required the Student’s *t*-test was used to determine significance of differences noted between control (DMSO treated lenses) and drug treated lenses. Values are provided as Mean ± S.E.M, and a *P*<0.05 was considered statistically significant for all comparisons.

## Supporting Information

Figure S1 **Cell viability and cytotoxicity in mouse lens epithelial cells treated with felodipine.** To evaluate felodipine-induced effects on cell viability and cytotoxicity, mouse lens primary epithelial cells were grown on gelatin-coated plastic dishes, serum starved overnight and treated with felodipine (10 µM) for 6 or 24 hrs. Following this, cells were rinsed with DMEM and treated with fluorescein diacetate (2 µg/well) and propedium iodide (0.6 µg/well) for 3 min prior to imaging the green fluorescence derived from in vivo fluorescein diacetate hydrolysis and propidium iodide red nuclei staining in live cells. As shown in the figure, both control and felodipine treated cells were found to exhibit comparable viability and fluorescein diacetate-based green fluorescence. Additionally, there was no propidium iodide incorporation into cell nuclei in either the drug treated or the control cells, confirming absence of drug-induced cytotoxic effects. Scale bar indicates image magnification. The images are representative of triplicate analyses.
(TIF)

## References

[1] Bassnett S, Shi Y, Vrensen GF (2011) Biological glass: structural determinants of eye lens transparency. Philos Trans R Soc Lond B Biol Sci 366: 1250–1264. 21402584 10.1098/rstb.2010.0302 PMC3061108

[2] Mathias RT, Kistler J, Donaldson P (2007) The lens circulation. J Membr Biol 216: 1–16. 17568975 10.1007/s00232-007-9019-y

[3] Lovicu FJ, McAvoy JW (2005) Growth factor regulation of lens development. Dev Biol 280: 1–14. 15766743 10.1016/j.ydbio.2005.01.020

[4] Song S, Landsbury A, Dahm R, Liu Y, Zhang Q, et al (2009) Functions of the intermediate filament cytoskeleton in the eye lens. J Clin Invest 119: 1837–1848. 19587458 10.1172/JCI38277 PMC2701874

[5] Mathias RT, White TW, Gong X (2010) Lens gap junctions in growth, differentiation, and homeostasis. Physiol Rev 90: 179–206. 20086076 10.1152/physrev.00034.2009 PMC4627646

[6] Wistow GJ, Piatigorsky J (1988) Lens crystallins: the evolution and expression of proteins for a highly specialized tissue. Annu Rev Biochem 57: 479–504. 3052280 10.1146/annurev.bi.57.070188.002403

[7] Maddala R, Skiba NP, Lalane R, 3rd, Sherman DL, Brophy PJ, et al (2011) Periaxin is required for hexagonal geometry and membrane organization of mature lens fibers. Dev Biol 357: 179–190. 21745462 10.1016/j.ydbio.2011.06.036 PMC3164832

[8] Straub BK, Boda J, Kuhn C, Schnoelzer M, Korf U, et al (2003) A novel cell-cell junction system: the cortex adhaerens mosaic of lens fiber cells. J Cell Sci 116: 4985–4995. 14625392 10.1242/jcs.00815

[9] Wang Z, Schey KL (2011) Aquaporin-0 interacts with the FERM domain of ezrin/radixin/moesin proteins in the ocular lens. Invest Ophthalmol Vis Sci 52: 5079–5087. 21642618 10.1167/iovs.10-6998 PMC3176042

[10] More MI, Kirsch FP, Rathjen FG (2001) Targeted ablation of NrCAM or ankyrin-B results in disorganized lens fibers leading to cataract formation. J Cell Biol 154: 187–196. 11449000 10.1083/jcb.200104038 PMC2196853

[11] Ogawa Y, Rasband MN (2008) The functional organization and assembly of the axon initial segment. Curr Opin Neurobiol 18: 307–313. 18801432 10.1016/j.conb.2008.08.008

[12] Salzer JL, Brophy PJ, Peles E (2008) Molecular domains of myelinated axons in the peripheral nervous system. Glia 56: 1532–1540. 18803321 10.1002/glia.20750

[13] Sherman DL, Brophy PJ (2005) Mechanisms of axon ensheathment and myelin growth. Nat Rev Neurosci 6: 683–690. 16136172 10.1038/nrn1743

[14] Bennett V, Healy J (2009) Membrane domains based on ankyrin and spectrin associated with cell-cell interactions. Cold Spring Harb Perspect Biol 1: a003012. 20457566 10.1101/cshperspect.a003012 PMC2882121

[15] Alvarez JL, Petzhold D, Pankonien I, Behlke J, Kouno M, et al (2010) Ahnak1 modulates L-type Ca(2+) channel inactivation of rodent cardiomyocytes. Pflugers Arch 460: 719–730. 20607281 10.1007/s00424-010-0853-x

[16] Matza D, Flavell RA (2009) Roles of Ca(v) channels and AHNAK1 in T cells: the beauty and the beast. Immunol Rev 231: 257–264. 19754902 10.1111/j.1600-065X.2009.00805.x

[17] Pankonien I, Alvarez JL, Doller A, Kohncke C, Rotte D, et al (2011) Ahnak1 is a tuneable modulator of cardiac Ca(v)1.2 calcium channel activity. J Muscle Res Cell Motil 32: 281–290. 22038483 10.1007/s10974-011-9269-2

[18] Shao Y, Czymmek KJ, Jones PA, Fomin VP, Akanbi K, et al (2009) Dynamic interactions between L-type voltage-sensitive calcium channel Cav1.2 subunits and ahnak in osteoblastic cells. Am J Physiol Cell Physiol 296: C1067–1078. 19261907 10.1152/ajpcell.00427.2008 PMC2681378

[19] Duncan G, Collison DJ (2002) Calcium signalling in ocular tissues: functional activity of G-protein and tyrosine-kinase coupled receptors. Exp Eye Res 75: 377–389. 12387785

[20] Rhodes JD, Sanderson J (2009) The mechanisms of calcium homeostasis and signalling in the lens. Exp Eye Res 88: 226–234. 19061888 10.1016/j.exer.2008.10.025

[21] Duncan G, Jacob TJ (1984) Calcium and the physiology of cataract. Ciba Found Symp 106: 132–152. 6096095 10.1002/9780470720875.ch8

[22] Truscott RJ, Marcantonio JM, Tomlinson J, Duncan G (1990) Calcium-induced opacification and proteolysis in the intact rat lens. Invest Ophthalmol Vis Sci 31: 2405–2411. 2173688

[23] Duncan G, Wormstone IM (1999) Calcium cell signalling and cataract: role of the endoplasmic reticulum. Eye (Lond) 13 (Pt 3b): 480–483. 10.1038/eye.1999.125 10627828

[24] Sanderson J, Marcantonio JM, Duncan G (2000) A human lens model of cortical cataract: Ca2+-induced protein loss, vimentin cleavage and opacification. Invest Ophthalmol Vis Sci 41: 2255–2261. 10892870

[25] Nakamura Y, Fukiage C, Shih M, Ma H, David LL, et al (2000) Contribution of calpain Lp82-induced proteolysis to experimental cataractogenesis in mice. Invest Ophthalmol Vis Sci 41: 1460–1466. 10798663

[26] Shearer TR, Ma H, Fukiage C, Azuma M (1997) Selenite nuclear cataract: review of the model. Mol Vis 3: 8. 9238097

[27] Gao J, Sun X, Martinez-Wittinghan FJ, Gong X, White TW, et al (2004) Connections between connexins, calcium, and cataracts in the lens. J Gen Physiol 124: 289–300. 15452195 10.1085/jgp.200409121 PMC2233908

[28] Girsch SJ, Peracchia C (1991) Calmodulin interacts with a C-terminus peptide from the lens membrane protein MIP26. Curr Eye Res 10: 839–849. 1790714 10.3109/02713689109013880

[29] Gold MG, Reichow SL, O’Neill SE, Weisbrod CR, Langeberg LK, et al (2012) AKAP2 anchors PKA with aquaporin-0 to support ocular lens transparency. EMBO Mol Med 4: 15–26. 22095752 10.1002/emmm.201100184 PMC3272850

[30] Lampe PD, Bazzi MD, Nelsestuen GL, Johnson RG (1986) Phosphorylation of lens intrinsic membrane proteins by protein kinase C. Eur J Biochem. 156: 351–357. 10.1111/j.1432-1033.1986.tb09590.x 2422029

[31] Lurtz MM, Louis CF (2003) Calmodulin and protein kinase C regulate gap junctional coupling in lens epithelial cells. Am J Physiol Cell Physiol 285: C1475–1482. 12917107 10.1152/ajpcell.00361.2002

[32] Nemeth-Cahalan KL, Hall JE (2000) pH and calcium regulate the water permeability of aquaporin 0. J Biol Chem 275: 6777–6782. 10702234 10.1074/jbc.275.10.6777

[33] Nemeth-Cahalan KL, Kalman K, Hall JE (2004) Molecular basis of pH and Ca2+ regulation of aquaporin water permeability. J Gen Physiol 123: 573–580. 15078916 10.1085/jgp.200308990 PMC2234493

[34] Rose KM, Wang Z, Magrath GN, Hazard ES, Hildebrandt JD, et al (2008) Aquaporin 0-calmodulin interaction and the effect of aquaporin 0 phosphorylation. Biochemistry 47: 339–347. 18081321 10.1021/bi701980t

[35] Varadaraj K, Kumari S, Shiels A, Mathias RT (2005) Regulation of aquaporin water permeability in the lens. Invest Ophthalmol Vis Sci 46: 1393–1402. 15790907 10.1167/iovs.04-1217

[36] Welsh MJ, Aster JC, Ireland M, Alcala J, Maisel H (1982) Calmodulin binds to chick lens gap junction protein in a calcium-independent manner. Science 216: 642–644. 6280283 10.1126/science.6280283

[37] Aravind P, Mishra A, Suman SK, Jobby MK, Sankaranarayanan R, et al (2009) The betagamma-crystallin superfamily contains a universal motif for binding calcium. Biochemistry 48: 12180–12190. 19921810 10.1021/bi9017076

[38] Cengiz M, Gurkaynak M, Atahan IL, Kilic K, Totan Y (1999) The effect of verapamil in the prevention of radiation-induced cataract. Int J Radiat Oncol Biol Phys 43: 623–626. 10078648 10.1016/s0360-3016(98)00458-1

[39] Ettl A, Daxer A, Gottinger W, Schmid E (2004) Inhibition of experimental diabetic cataract by topical administration of RS-verapamil hydrochloride. Br J Ophthalmol 88: 44–47. 14693771 10.1136/bjo.88.1.44 PMC1771934

[40] Kametaka S, Kasahara T, Ueo M, Takenaka M, Saito M, et al (2008) Effect of nifedipine on severe experimental cataract in diabetic rats. J Pharmacol Sci 106: 651–658. 18431041 10.1254/jphs.fp0072294

[41] Pierce GN, Afzal N, Kroeger EA, Lockwood MK, Kutryk MJ, et al (1989) Cataract formation is prevented by administration of verapamil to diabetic rats. Endocrinology 125: 730–735. 2752974 10.1210/endo-125-2-730

[42] Maddala R, Skiba N, Vasantha Rao P (2007) Lens fiber cell elongation and differentiation is associated with a robust increase in myosin light chain phosphorylation in the developing mouse. Differentiation 75: 713–725. 17459090 10.1111/j.1432-0436.2007.00173.x

[43] Striessnig J, Bolz HJ, Koschak A (2010) Channelopathies in Cav1.1, Cav1.3, and Cav1.4 voltage-gated L-type Ca2+ channels. Pflugers Arch 460: 361–374. 20213496 10.1007/s00424-010-0800-x PMC2883925

[44] Tsien RW, Ellinor PT, Horne WA (1991) Molecular diversity of voltage-dependent Ca2+ channels. Trends Pharmacol Sci 12: 349–354. 1659003 10.1016/0165-6147(91)90595-j

[45] Catterall WA (2011) Voltage-gated calcium channels. Cold Spring Harb Perspect Biol 3: a003947. 21746798 10.1101/cshperspect.a003947 PMC3140680

[46] Catterall WA, Perez-Reyes E, Snutch TP, Striessnig J (2005) International Union of Pharmacology. XLVIII. Nomenclature and structure-function relationships of voltage-gated calcium channels. Pharmacol Rev 57: 411–425. 16382099 10.1124/pr.57.4.5

[47] Rampe D, Triggle DJ (1990) New ligands for L-type Ca2+ channels. Trends Pharmacol Sci 11: 112–115. 2104462 10.1016/0165-6147(90)90196-f

[48] Jiang JX (2010) Gap junctions or hemichannel-dependent and independent roles of connexins in cataractogenesis and lens development. Curr Mol Med 10: 851–863. 21091421 10.2174/156652410793937750 PMC6263138

[49] Beguin P, Mahalakshmi RN, Nagashima K, Cher DH, Takahashi A, et al (2005) 14-3-3 and calmodulin control subcellular distribution of Kir/Gem and its regulation of cell shape and calcium channel activity. J Cell Sci 118: 1923–1934. 15860732 10.1242/jcs.02321

[50] Czirjak G, Vuity D, Enyedi P (2008) Phosphorylation-dependent binding of 14-3-3 proteins controls TRESK regulation. J Biol Chem 283: 15672–15680. 18397886 10.1074/jbc.M800712200 PMC3259650

[51] Li Y, Wu Y, Zhou Y (2006) Modulation of inactivation properties of CaV2.2 channels by 14-3-3 proteins. Neuron 51: 755–771. 16982421 10.1016/j.neuron.2006.08.014

[52] Zuzarte M, Heusser K, Renigunta V, Schlichthorl G, Rinne S, et al (2009) Intracellular traffic of the K+ channels TASK-1 and TASK-3: role of N- and C-terminal sorting signals and interaction with 14-3-3 proteins. J Physiol 587: 929–952. 19139046 10.1113/jphysiol.2008.164756 PMC2673767

[53] Liang X, Da Paula AC, Bozoky Z, Zhang H, Bertrand CA, et al (2012) Phosphorylation-dependent 14-3-3 protein interactions regulate CFTR biogenesis. Mol Biol Cell 23: 996–1009. 22278744 10.1091/mbc.E11-08-0662 PMC3302758

[54] Liang X, Butterworth MB, Peters KW, Walker WH, Frizzell RA (2008) An obligatory heterodimer of 14-3-3beta and 14-3-3epsilon is required for aldosterone regulation of the epithelial sodium channel. J Biol Chem 283: 27418–27425. 18687683 10.1074/jbc.M803687200 PMC2562081

[55] Somlyo AP, Somlyo AV (2003) Ca2+ sensitivity of smooth muscle and nonmuscle myosin II: modulated by G proteins, kinases, and myosin phosphatase. Physiol Rev 83: 1325–1358. 14506307 10.1152/physrev.00023.2003

[56] Clapham DE (2007) Calcium signaling. Cell 131: 1047–1058. 18083096 10.1016/j.cell.2007.11.028

[57] Ghosh A, Greenberg ME (1995) Calcium signaling in neurons: molecular mechanisms and cellular consequences. Science 268: 239–247. 7716515 10.1126/science.7716515

[58] Anderson ME (2004) Calmodulin kinase and L-type calcium channels; a recipe for arrhythmias? Trends Cardiovasc Med 14: 152–161. 15177266 10.1016/j.tcm.2004.02.005

[59] Dolmetsch RE, Pajvani U, Fife K, Spotts JM, Greenberg ME (2001) Signaling to the nucleus by an L-type calcium channel-calmodulin complex through the MAP kinase pathway. Science 294: 333–339. 11598293 10.1126/science.1063395

[60] Dai S, Hall DD, Hell JW (2009) Supramolecular assemblies and localized regulation of voltage-gated ion channels. Physiol Rev 89: 411–452. 19342611 10.1152/physrev.00029.2007 PMC2733249

[61] Trautwein W, Hescheler J (1990) Regulation of cardiac L-type calcium current by phosphorylation and G proteins. Annu Rev Physiol 52: 257–274. 2158764 10.1146/annurev.ph.52.030190.001353

[62] van Heyningen R, Harding JJ (1986) Do aspirin-like analgesics protect against cataract? A case-control study. Lancet 1: 1111–1113. 2871377 10.1016/s0140-6736(86)91834-9

[63] Harding JJ, van Heyningen R (1988) Drugs, including alcohol, that act as risk factors for cataract, and possible protection against cataract by aspirin-like analgesics and cyclopenthiazide. Br J Ophthalmol 72: 809–814. 3207655 10.1136/bjo.72.11.809 PMC1041596

[64] Cekic O, Bardak Y (1998) Lenticular calcium, magnesium, and iron levels in diabetic rats and verapamil effect. Ophthalmic Res 30: 107–112. 9523289 10.1159/000055462

[65] Devamanoharan PS, Varma SD (1995) Inhibition of polyol formation in rat lens by verapamil. J Ocul Pharmacol Ther 11: 527–531. 8574816 10.1089/jop.1995.11.527

[66] Meissner A, Noack T (2008) Proliferation of human lens epithelial cells (HLE-B3) is inhibited by blocking of voltage-gated calcium channels. Pflugers Arch 457: 47–59. 18446362 10.1007/s00424-008-0514-5

[67] Kinoshita JH (1974) Mechanisms initiating cataract formation. Proctor Lecture. Invest Ophthalmol 13: 713–724. 4278188

[68] Reichow SL, Gonen T (2008) Noncanonical binding of calmodulin to aquaporin-0: implications for channel regulation. Structure 16: 1389–1398. 18786401 10.1016/j.str.2008.06.011 PMC2605016

[69] Liu J, Xu J, Gu S, Nicholson BJ, Jiang JX (2011) Aquaporin 0 enhances gap junction coupling via its cell adhesion function and interaction with connexin 50. J Cell Sci 124: 198–206. 21172802 10.1242/jcs.072652 PMC3010190

[70] Yu XS, Jiang JX (2004) Interaction of major intrinsic protein (aquaporin-0) with fiber connexins in lens development. J Cell Sci 117: 871–880. 14762116 10.1242/jcs.00945

[71] Lichtstein D, McGowan MH, Russell P, Carper DA (2000) Digitalis and digitalislike compounds down-regulate gene expression of the intracellular signaling protein 14-3-3 in rat lens. Hypertens Res 23 Suppl: S51–53 10.1291/hypres.23.supplement_s51 11016820

[72] McGowan MH, Russell P, Carper DA, Lichtstein D (1999) Na+, K+-ATPase inhibitors down-regulate gene expression of the intracellular signaling protein 14-3-3 in rat lens. J Pharmacol Exp Ther 289: 1559–1563. 10336553

[73] Nguyen TA, Takemoto LJ, Takemoto DJ (2004) Inhibition of gap junction activity through the release of the C1B domain of protein kinase Cgamma (PKCgamma) from 14-3-3: identification of PKCgamma-binding sites. J Biol Chem 279: 52714–52725. 15459208 10.1074/jbc.M403040200

[74] Hund TJ, Koval OM, Li J, Wright PJ, Qian L, et al (2010) A beta(IV)-spectrin/CaMKII signaling complex is essential for membrane excitability in mice. J Clin Invest 120: 3508–3519. 20877009 10.1172/JCI43621 PMC2947241

[75] Seri M, Pecci A, Di Bari F, Cusano R, Savino M, et al (2003) MYH9-related disease: May-Hegglin anomaly, Sebastian syndrome, Fechtner syndrome, and Epstein syndrome are not distinct entities but represent a variable expression of a single illness. Medicine (Baltimore) 82: 203–215. 12792306 10.1097/01.md.0000076006.64510.5c

[76] Zhang Y, Conti MA, Malide D, Dong F, Wang A, et al (2012) Mouse models of MYH9-related disease: mutations in nonmuscle myosin II-A. Blood 119: 238–250. 21908426 10.1182/blood-2011-06-358853 PMC3251230

[77] Rao PV, Maddala R (2009) Abundant expression of ponsin, a focal adhesion protein, in lens and downregulation of its expression by impaired cytoskeletal signaling. Invest Ophthalmol Vis Sci 50: 1769–1777. 19029030 10.1167/iovs.08-2909 PMC2716002

[78] Ray R, de Ridder GG, Eu JP, Paton AW, Paton JC, et al (2012) The Escherichia coli subtilase cytotoxin A subunit specifically cleaves cell-surface GRP78 protein and abolishes COOH-terminal-dependent signaling. J Biol Chem 287: 32755–32769. 22851173 10.1074/jbc.M112.399808 PMC3463347

[79] Maddala R, Chauhan BK, Walker C, Zheng Y, Robinson ML, et al (2011) Rac1 GTPase-deficient mouse lens exhibits defects in shape, suture formation, fiber cell migration and survival. Dev Biol 360: 30–43. 21945075 10.1016/j.ydbio.2011.09.004 PMC3215831

