# Supplementary figures and images for: L-Type Calcium Channels Play a Critical Role in Maintaining Lens Transparency by Regulating Phosphorylation of Aquaporin-0 and Myosin Light Chain and Expression of Connexins
Source: PLoS One. 2013 May 29;8(5):e64676. doi: 10.1371/journal.pone.0064676 (PMC3667166; doi:10.1371/journal.pone.0064676)

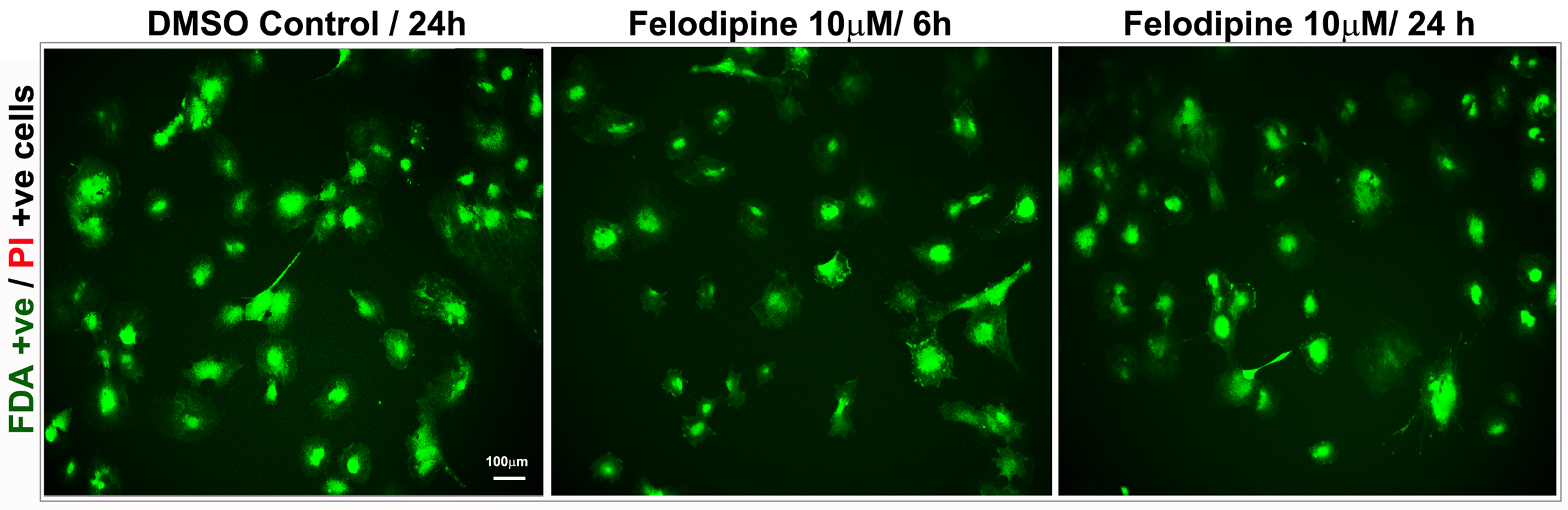

Supplement: Figure S1 — Cell viability and cytotoxicity in mouse lens epithelial cells treated with felodipine. To evaluate felodipine-induced effects on cell viability and cytotoxicity, mouse lens primary epithelial cells were grown on gelatin-coated plastic dishes, serum starved overnight and treated with felodipine (10 µM) for 6 or 24 hrs. Following this, cells were rinsed with DMEM and treated with fluorescein diacetate (2 µg/well) and propedium iodide (0.6 µg/well) for 3 min prior to imaging the green fluorescence derived from in vivo fluorescein diacetate hydrolysis and propidium iodide red nuclei staining in live cells. As shown in the figure, both control and felodipine treated cells were found to exhibit comparable viability and fluorescein diacetate-based green fluorescence. Additionally, there was no propidium iodide incorporation into cell nuclei in either the drug treated or the control cells, confirming absence of drug-induced cytotoxic effects. Scale bar indicates image magnification. The images are representative of triplicate analyses. (TIF) [file pone.0064676.s001.tif]
